# Comparative analysis of serum proteome in congenital scoliosis patients with *TBX6* haploinsufficiency – a first report pointing to lipid metabolism

**DOI:** 10.1111/jcmm.13341

**Published:** 2017-09-25

**Authors:** Qiankun Zhu, Nan Wu, Gang Liu, Yangzhong Zhou, Sen Liu, Jun Chen, Jiaqi Liu, Yuzhi Zuo, Zhenlei Liu, Weisheng Chen, Yixin Chen, Jia Chen, Mao Lin, Yanxue Zhao, Yang Yang, Shensgru Wang, Xu Yang, Yufen Ma, Jian Wang, Xiaoli Chen, Jianguo Zhang, Jianxiong Shen, Zhihong Wu, Guixing Qiu

**Affiliations:** ^1^ Department of Orthopedic Surgery Peking Union Medical College Hospital Peking Union Medical College and Chinese Academy of Medical Sciences Beijing China; ^2^ Beijing Key Laboratory for Genetic Research of Skeletal Deformity Beijing China; ^3^ Research Center of Orthopedics/Rare Disease Chinese Academy of Medical Sciences Beijing China; ^4^ Tsinghua University Medical School Beijing China; ^5^ Department of Pathology Beijing Ditan Hospital Capital Medical University Beijing China; ^6^ Department of Neurosurgery Xuanwu Hospital Capital Medical University Beijing China; ^7^ Department of Medical Genetics Molecular Diagnostic Laboratory Shanghai Children's Medical Center Shanghai Jiaotong University School of Medicine Shanghai China; ^8^ Department of Medical Genetics Beijing Municipal Key Laboratory of Child Development and Nutriomics Capital Institute of Pediatrics Beijing China; ^9^ Department of Central Laboratory Peking Union Medical College Hospital Peking Union Medical College and Chinese Academy of Medical Sciences Beijing China

**Keywords:** congenital scoliosis, *TBX6*, proteomics, isobaric tagged relative and absolute quantification, lipid metabolism

## Abstract

Congenital scoliosis (CS) is a three‐dimensional deformity of the spine affecting quality of life. We have demonstrated *TBX6* haploinsufficiency is the most important contributor to CS. However, the pathophysiology at the protein level remains unclear. Therefore, this study was to explore the differential proteome in serum of CS patients with *TBX6* haploinsufficiency. Sera from nine CS patients with *TBX6* haploinsufficiency and nine age‐ and gender‐matched healthy controls were collected and analysed by isobaric tagged relative and absolute quantification (iTRAQ) labelling coupled with mass spectrometry (MS). In total, 277 proteins were detected and 20 proteins were designated as differentially expressed proteins, which were submitted to subsequent bioinformatics analysis. Gene Ontology classification analysis showed the biological process was primarily related to ‘cellular process’, molecular function ‘structural molecule activity’ and cellular component ‘extracellular region’. IPA analysis revealed ‘LXR/RXR activation’ was the top pathway, which is a crucial pathway in lipid metabolism. Hierarchical clustering analysis generated two clusters. In summary, this study is the first proteomic research to delineate the total and differential serum proteins in *TBX6* haploinsufficiency‐caused CS. The proteins discovered in this experiment may serve as potential biomarkers for CS, and lipid metabolism might play important roles in the pathogenesis of CS.

## Introduction

CS is a complex three‐dimensional deformity of the spine with a lateral curvature of more than ten degrees 1. The overall incidence of CS has been estimated at approximately one of 2000 live births 2. CS originates from defects of the axial skeletal development during embryogenesis. Disruption of normal somitogenesis in the embryonic period which is regulated by a multitude of genes can result in vertebral malformations 3. If left untreated, the scoliotic deformity of the CS, specifically in the thoracic region, can progress to serious conditions devastating the pulmonary function, hence augmenting the mortality 4, 5.

The T‐box genes are an evolutionarily conserved family of genes encoding transcription factors that play a key role in regulating the development processes 6. The T‐box gene *TBX6*, located at the 16p11.2 region, with 6092 bp in size, is accountable for normal somitogenesis by interacting with the Notch pathway in the clock‐wavefront model 7, 8. More recently, our team reported that *TBX6* haploinsufficiency is associated with sporadic cases of CS 1. Involvement of *TBX6* haploinsufficiency in CS cases is endorsed by the identification of *TBX6* null mutation in conjunction with a hypomorphic allele to account for 8‐11% of CS cases 1. To date, the *TBX6* compound inheritance model has shed light on the maximum ratio of CS 9, suggesting *TBX6* one of the most important genes in the occurrence and pathogenesis of CS.

Although the involvement of *TBX6* gene in CS has been well identified, further biological researches are needed to clarify the exact mechanisms of *TBX6* in CS from more aspects. While gene is the carrier for genetic information, protein is the final output of gene and the executor of exact biological functions 10, 11. In this regard, it is quite compelling to elucidate the role of *TBX6* in CS from the protein perspective. One desirable way is to utilize the proteomics technique to detect the total proteins, namely the global proteome profiles, thus complementing our previous findings on *TBX6*. However, to the best of our knowledge, there are no publications using proteomics techniques to study the CS disease. Therefore, this study is conceived to establish the serum proteome profiles of CS cases with *TBX6* haploinsufficiency by iTRAQ technique.

## Materials and methods

### Patients and sample obtainment

In the previous genetic experiment, genome‐wide analyses of copy number variants were performed by Agilent oligonucleotide comparative genomic hybridization (CGH) microarrays. Additionally, a custom 4 × 180k CGH microarray with approximately 180,000 oligonucleotide probes was utilized. DNA processing and microarray experiment were following the Agilent oligonucleotide CGH instruction (Protocol version 6.0). Quantitative PCR analysis was conducted through ∆Ct method using SYBR Green Realtime PCR Master Mix (TOYOBO, Osaka, Japan) 1.

As for the proteomic experiment, a case–control study was adopted. The sera of the nine patients and nine healthy controls were drawn from the previous cohort. The exclusion criteria were exactly the same as previously mentioned 1, in which known syndromes (including Alagille syndrome, Goldenhar's syndrome, hemifacial microsomia, Klippel–Feil syndrome, spondylocostal dysostosis, spondylothoracic dysostosis and VACTERL syndrome) were excluded clinically. Herein, the inclusion criteria for case group were as follows: (*i*) clinical diagnosis of CS; and (*ii*) carrying a genotype of 16p11.2 deletion and a common *TBX6* haplotype of TCA (defined by the non‐reference alleles of three common single‐nucleotide polymorphisms [SNPs]‐rs2289292, rs3809624 and rs3809627, respectively). Age and gender were matched between the case and control groups to guarantee there was no statistical significance. This work had been approved by the ethical committee of Peking Union Medical College Hospital, and written informed consent had been obtained from the participants or their guardians.

### Depletion of high‐abundance serum proteins

Serum IgG and albumin were depleted using ProteoPrep Blue Albumin and IgG Depletion kit (PROTBA, Sigma‐Aldrich Company, Darmstadt, Germany), according to the manufacturer's instruction. A total of 600 μl serum was used for the depletion, and finally, 400 μl was eluted at the end of the procedure. The protein concentration after depletion was evaluated by Bradford method.

### Trypsin digestion and iTRAQ labelling

Peptides from 100 μg of each depleted serum were labelled with 8‐plex iTRAQ reagents (Applied Biosystems, Foster City, CA, USA), according to the manufacturer's protocol. In short, the proteins were denatured, alkylated and trypsin‐digested at 37°C overnight. Then the peptides were labelled with one unit of iTRAQ reagent that was reconstituted in 150 μl isopropanol. The labelling arrangement was shown in Table 1. After the labelling, the samples were combined into one tube and dried in vacuo. Dried peptides were resuspended in 100 μl of mobile phase A and were centrifuged at 14,000 ***g*** for 20 min. The supernatants were loaded on the column (Durashell‐C18,4.6 × 250 mm, 5 μm, 100 Å, Agela, DC952505‐0) and eluted stepwise by injecting mobile B in the RIGOL L‐3000 system (RIGOL, Beijing, China). Mobile phase A consisted of 2% (v/v) acetonitrile, 98% (v/v) ddH_2_O and pH 10, and phase B consisted of 98% (v/v) acetonitrile, 2% (v/v) ddH_2_O and pH 10. The 60‐min. gradients comprised 5% mobile B for 5 min., 5–30% mobile B for 35 min., 30–95% mobile B for 10 min. and equilibrated with 5% mobile B for 10 min. at a 300 nl/min. flow rate. The fractions were eluted at 1.5‐min. intervals and collected using step gradients of mobile B.

**Table 1:** Labelling design for samples from CS and control individuals. The digested peptides were labelled with 113–121 iTRAQ isotopes. A total of 100 μg proteins were labelled with 1 unit of label. Three replicates were performed for the pooled sample (121 isotopes). CS is the acronym of congenital scoliosis

	113	114	115	116	117	118	119	121	
1	CS 1	CS 2	CS 3	CS 4	Control 1	Control 2	Control 3	Pool	8‐plex
2	Control 4	Control 5	Control 6	Control 7	CS 5	CS 6	CS 7	Pool	8‐plex
3		CS 8	CS 9	Control 8	Control 9			Pool	8‐plex

### Mass spectrometer analysis

LC‐MS/MS analysis was performed on Q‐Exactive mass spectrometer (Thermo Fisher Scientific, Waltham, MA, USA) equipped with a nano‐liquid chromatography system (Thermo Scientific EASY‐nLC 1000 System, Waltham, MA, USA). The dried iTRAQ‐labelled peptides were dissolved in 20 μl of liquid containing 2% methanol and 0.1% formic acid. After centrifugation at 12,000 ***g*** for 10 min., the supernatant was collected. Then, peptide mixtures were separated using a binary solvent system with 99.9% H_2_O, 0.1% formic acid (phase A) and 99.9% acetonitrile, 0.1% formic acid (phase B). Linear gradients of 8–30% B for 77 min., 30–50% B for 10 min., 50–95% B for 15 min. and finally 5% B for 11 min., with a flow rate of 350 nl/min., were employed. The eluent was submitted to Q‐Exactive mass spectrometer through EASY‐Spray ion source with parameters as follows: spray voltage, 2.3 kv; capillary temperature, 320°C; and declustering potential, 100 V. The mass spectrometer was adjusted automatically between MS and MS/MS mode. The full‐scan MS mode was operated with the following parameters: automatic gain control (AGC) target, 3e6; maximum ion transfer, 20 ms; resolution, 70,000 FWHM; and scan range, 300–1800 m/z. The MS/MS mode was set as follows: AGC target, 1e5; maximum ion transfer, 120 ms; intensity threshold, 8.30E+03; fragmentation methods, high‐energy collisional dissociation (HCD); normalized collision energy, 29%; and top number, 20.

### Database searching and protein identification

We used ProteoWizard (version 3.0.8789) to extract the raw data files from MS. Charge state deconvolution and deisotoping were not performed. The MS/MS samples were searched using the Mascot search engine against the SwissProt database (2016_03, selected for Homo sapiens, unknown version, 20200 entries). The search was governed by the following parameters: trypsin was assumed as the digestion enzyme; two missed cleavages were allowed at maximum; fragment ion mass tolerance of 20 mmu; parent ion tolerance of 15 ppm; carbamidomethylation of cysteine was selected as fixed modification; oxidation of methionine, iTRAQ 8 plex of lysine and the N‐terminus were set as variable modifications. Protein quantification was performed by Scaffold Q+ (4.6.1, Proteome Software Inc., Portland, OR, USA) to calculate the relative abundance of iTRAQ‐labelled peptides and the corresponding proteins. Peptide identification was accepted at greater than 95% probability to achieve FDR < 1.0%. Proteins identifications were accepted if more than 96% probability to achieve FDR < 1.0%. Only the proteins with at least two identified peptides were considered for quantification. Protein probabilities were calculated by the Protein Prophet algorithm 12. Principle of parsimony was applied for grouping of proteins that contained similar peptides and could not be differentiated based on MS/MS analysis alone. Normalization calculation was performed with median protein ratio iteratively on intensities. Spectral data were log‐transformed, pruned of those that can match to multiple proteins and with a missing value.

### Bioinformatics analysis

The distribution description of the data in each group is generally presented as mean ± standard deviation (S.D.) The total proteins were submitted to Student's t‐tests to determine the significance between two groups by Perseus software (version 1.5.0.31, Berlin, Germany). The *q* values were calculated on the basis of permutation‐based FDR default setting in Perseus. Ratio is defined as 2^*difference*^, while difference equals the mean of CS log‐transformed spectral intensities minus the mean of control log‐transformed spectral intensities. The significance threshold of *q* values was 0.05. Ratio cut‐off values were set as 1.42 or 0.70. Differentially expressed proteins were designated when meeting the two requirements simultaneously: (*i*) *q* values < 0.05; and (*ii*) ratios ≥ 1.42 (up‐regulated) or ≤0.70 (down‐regulated). Gene Ontology (GO) analysis was performed on the PANTHER platform (http://www.pantherdb.org/), in which the biological process, molecular function and cellular component were analysed. The Ingenuity Pathway Analysis software (IPA^@^, v01‐04, QIANGEN Redwood City, CA, USA) was utilized to conduct the network and pathway analysis. In the IPA parameters, no expression cut‐off value was selected, and both up‐regulated and down‐regulated proteins were focused on. Hierarchical clustering analysis with Euclidean distance was carried out after Z‐score transformation, and K means pre‐processing by Perseus.

## Results

### Detection and identification of total proteins and differentially expressed proteins

**Figure 1:**
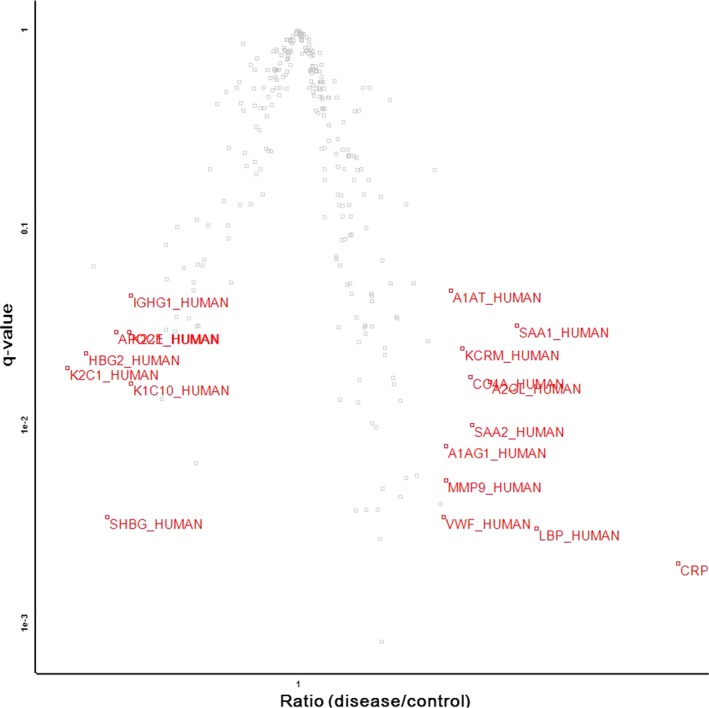
Volcano plot representation of totally identified protein results showing the ratio (*x*‐axis) and significance (*q* values, *y*‐axis). The volcano plot indicates that 20 proteins were significantly (*q* value < 0.05) differentially (ratio ≥1.42 or ≤0.70) expressed in the CS group compared with the normal control group. The accession numbers of each differentially expressed protein are shown in red.

In total, we identified 277 proteins that were expressing in both CS and control groups. Among the identified total proteins, 65 proteins showed a *q* value < 0.05. There are 108 up‐regulated (ratio > 1) and 169 down‐regulated proteins (ratio < 1) in the CS group in comparison with the control group, with ratio (CS/control) ranging from 0.573 to 2.052 (Table S1). Taking into account both the *q* value and the ratio, we designated 20 differentially expressed proteins (*q* < 0.05 and ratio ≥ 1.42 or ≤0.70) (Table 2)(Fig. 1). The differentially expressed proteins included apolipoprotein C‐I, sex hormone‐binding globulin, complement C4‐A and transgelin‐2.

**Table 2:** The 20 differentially expressed proteins in serum identified by LC‐MS/MS analysis in CS and normal control samples

Accession number	Protein name	CS mean	CS S.D.	Control mean	Control S.D.	Ratio (CS/control)	Up‐ or down‐regulation	−Log *P*‐value	*q* value	Differentially expressed proteins
CO9_HUMAN	Complement component C9 OS = Homo sapiens GN = C9 PE = 1 SV = 2	17.432	0.220	16.912	0.165	1.434	UP	4.463	0.000	+
TAGL2_HUMAN	Transgelin‐2 OS = Homo sapiens GN = TAGLN2 PE = 1 SV = 3	15.599	0.287	14.964	0.233	1.552	UP	4.014	0.000	+
CRP_HUMAN	C‐reactive protein OS = Homo sapiens GN = CRP PE = 1 SV = 1	16.620	0.801	15.297	0.210	2.502	UP	3.702	0.002	+
LBP_HUMAN	Lipopolysaccharide‐binding protein OS = Homo sapiens GN = LBP PE = 1 SV = 3	16.160	0.407	15.331	0.343	1.776	UP	3.593	0.003	+
SHBG_HUMAN	Sex hormone‐binding globulin OS = Homo sapiens GN = SHBG PE = 1 SV = 2	16.518	0.254	17.181	0.335	0.631	Down	3.643	0.003	+
VWF_HUMAN	Von Willebrand factor OS = Homo sapiens GN = VWF PE = 1 SV = 4	16.863	0.303	16.356	0.178	1.422	UP	3.293	0.003	+
MMP9_HUMAN	Matrix metalloproteinase‐9 OS = Homo sapiens GN = MMP9 PE = 1 SV = 3	15.666	0.313	15.151	0.216	1.428	UP	3.041	0.005	+
A1AG1_HUMAN	α‐1‐acid glycoprotein 1 OS = Homo sapiens GN = ORM1 PE = 1 SV = 1	17.388	0.269	16.872	0.289	1.430	UP	2.914	0.008	+
SAA2_HUMAN	Serum amyloid A‐2 protein OS = Homo sapiens GN = SAA2 PE = 1 SV = 1	17.382	0.453	16.777	0.169	1.522	UP	2.766	0.010	+
K1C10_HUMAN	Keratin, type I cytoskeletal 10 OS = Homo sapiens GN = KRT10 PE = 1 SV = 6	16.266	0.080	16.844	0.507	0.669	Down	2.422	0.016	+
A2GL_HUMAN	Leucine‐rich α‐2‐glycoprotein OS = Homo sapiens GN = LRG1 PE = 1 SV = 2	16.537	0.528	15.868	0.266	1.590	UP	2.430	0.017	+
CO4A_HUMAN	Complement C4‐A OS = Homo sapiens GN = C4A PE = 1 SV = 2	17.520	0.278	16.919	0.468	1.517	UP	2.357	0.018	+
K2C1_HUMAN	Keratin, type II cytoskeletal 1 OS = Homo sapiens GN = KRT1 PE = 1 SV = 6	16.021	0.171	16.823	0.721	0.573	Down	2.296	0.020	+
HBG2_HUMAN	Haemoglobin subunit γ‐2 OS = Homo sapiens GN = HBG2 PE = 1 SV = 2	15.672	0.503	16.409	0.482	0.600	Down	2.228	0.023	+
KCRM_HUMAN	Creatine kinase M‐type OS = Homo sapiens GN = CKM PE = 1 SV = 2	15.127	0.469	14.556	0.279	1.486	UP	2.198	0.024	+
APOC1_HUMAN	Apolipoprotein C‐I OS = Homo sapiens GN = APOC1 PE = 1 SV = 1	17.607	0.543	18.237	0.319	0.646	Down	2.074	0.029	+
K22E_HUMAN	Keratin, type II cytoskeletal 2 epidermal OS = Homo sapiens GN = KRT2 PE = 1 SV = 2	14.629	0.233	15.213	0.540	0.667	Down	2.055	0.030	+
SAA1_HUMAN	Serum amyloid A‐1 protein OS = Homo sapiens GN = SAA1 PE = 1 SV = 1	16.743	0.756	15.982	0.225	1.695	UP	1.977	0.032	+
IGHG1_HUMAN	Ig γ‐1 chain C region OS = Homo sapiens GN = IGHG1 PE = 1 SV = 1	16.583	0.548	17.162	0.350	0.669	Down	1.777	0.045	+
A1AT_HUMAN	α‐1‐anti‐trypsin OS = Homo sapiens GN = SERPINA1 PE = 1 SV = 3	17.018	0.559	16.487	0.235	1.445	UP	1.737	0.048	+

The spectral intensity was log‐transformed and compared by Student's t‐test with permutation‐based FDR test using Perseus. The significance cut‐off of *q* values was 0.05, and ratio cut‐off values were 1.42 or 0.70. Differentially expressed proteins were assigned if ratio ≥1.42 or ≤0.70 and *q* values < 0.05.

### GO classification of identified proteins

**Figure 2:**
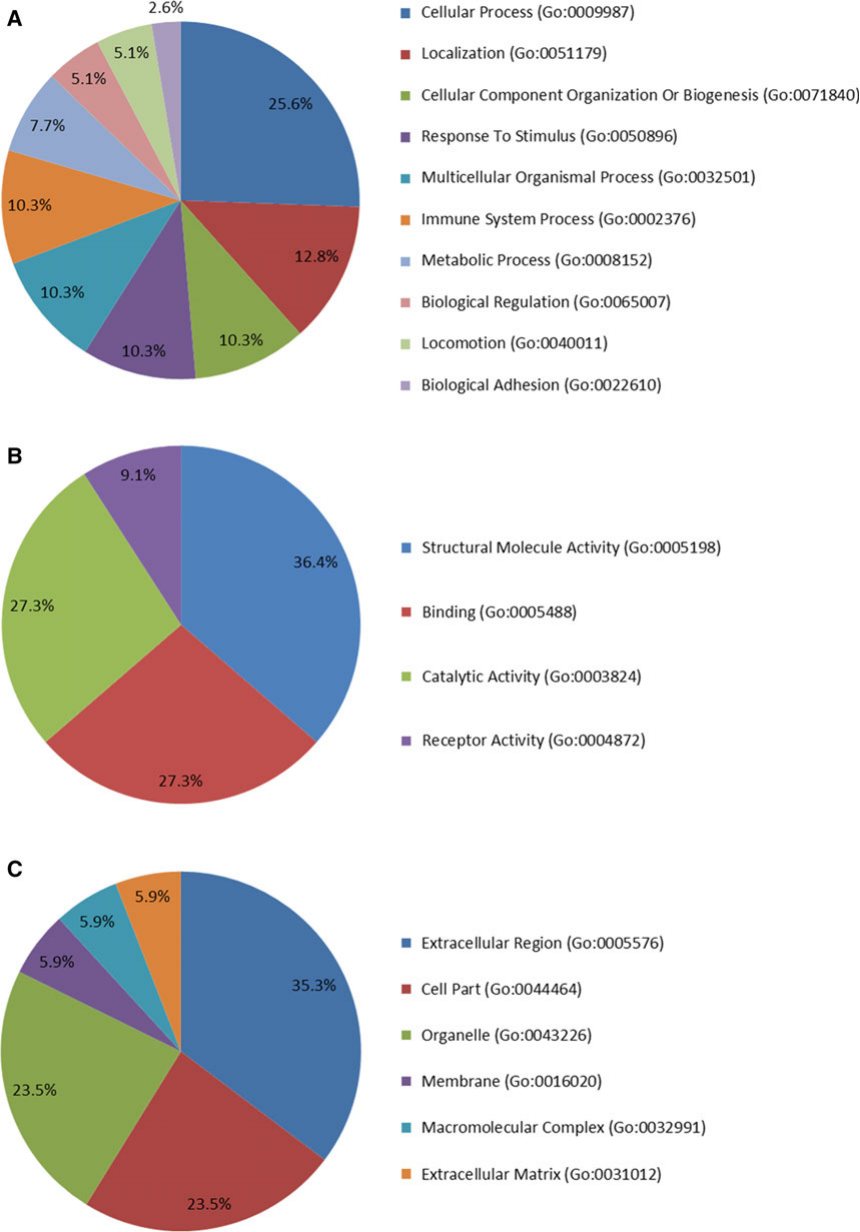
Classifications of the identified differential proteins according to GO annotation of biological process (**A**), molecular function (**B**) and cellular component (**C**). The GO analysis was performed on the PANTHER platform, and a total of 20 differentially expressed proteins were submitted to the analysis.

We classified the 20 differentially expressed proteins according to GO information (PANTHER platform, http://www.pantherdb.org/) concerning biological process, molecular function and cellular component. The biological processes of the 20 proteins are related to cellular process (25.6%), localization (12.8%), cellular component organization or biogenesis (10.3%), response to stimulus (10.3%), multicellular organismal process (10.3%), immune system process (10.3%), metabolic process (7.7%), biological regulation (5.1%), locomotion (5.1%) and biological adhesion (2.6%) (Fig. 2A). The top one molecular function is the structural molecule activity (36.4%); other molecular functions are involved in: binding (27.3%), catalytic activity (27.3%) and receptor activity (9.1%) (Fig. 2B). The cellular component analysis revealed that the main cellular component categories of these proteins are extracellular region (35.3%), cell part (23.5%), organelle (23.5%), membrane (5.9%), macromolecular complex (5.9%) and extracellular matrix (5.9%) (Fig. 2C).

### IPA network and pathway analysis

**Figure 3:**
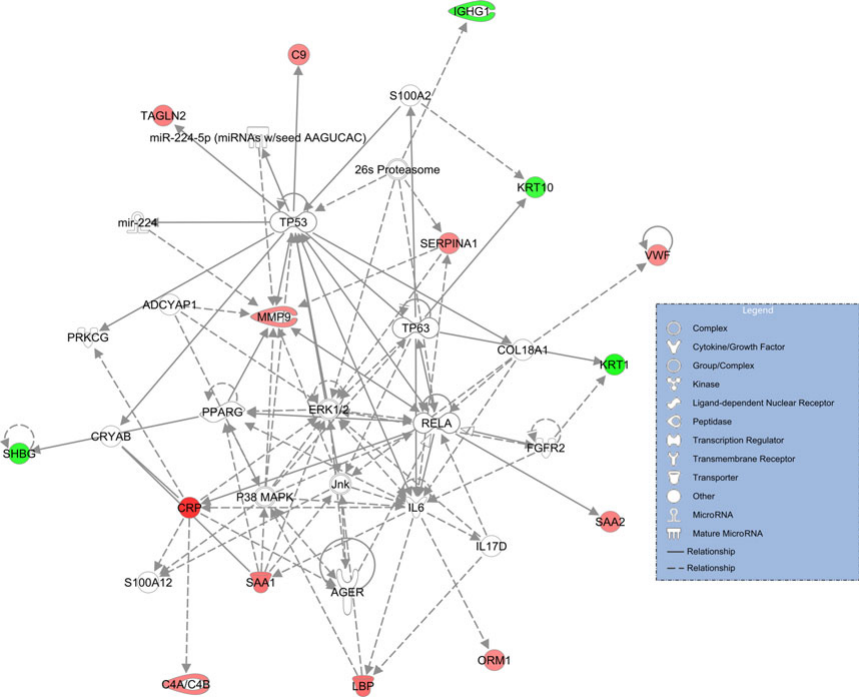
The top molecular network generated by IPA software for 20 differentially expressed proteins in serum of CS *versus* control. The modulated proteins are classified by established relationships into the functional analysis of a network. Solid line indicates direct interaction, and dashed line means indirect interaction.

Network analyses of 20 differentially expressed proteins in the serum of CS samples *versus* controls were assessed through the use of IPA software. The IPA calculates the *P*‐values by Fisher's exact test and the p score $\left(p\;\text{score}=-{\text{log}}_{10}^{P\mathrm{value}}\right)$ to assess the probability of matching the submitted proteins in a protein–protein interaction by random chance. Generally, we found a total of four significant molecular networks (Table S2). The first remarkable networks are depicted in Figure 3. The first network with the highest score (37) consists of 15 ‘focus molecules’, and its related diseases and functions incorporate cellular movement, haematological system development and function and immune cell trafficking (Table S2).

**Figure 4:**
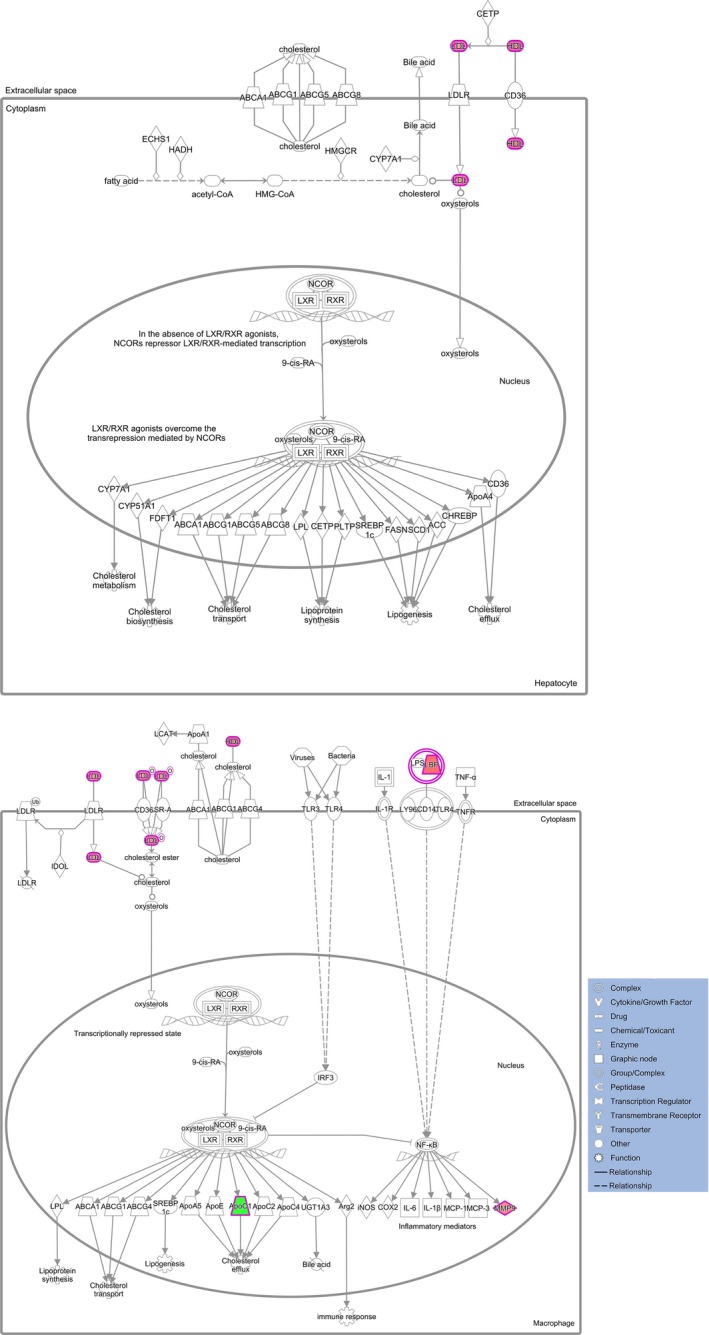
The top canonical pathway assigned by the IPA for 20 differentially expressed proteins expressed in both CS and normal control. The first canonical pathway of LXR/RXR activation. Each line and arrow suggests known functional or physical interaction. Red and green colours in each shape mean the up‐ or down‐regulation of pathway‐associated protein expression. Solid line indicates direct interaction, and dashed line means indirect interaction.

The canonical pathways assigned by IPA which involve the 20 differentially expressed proteins indicated that the top seven pathways are LXR/RXR activation, acute phase response signalling, FXR/RXR activation, atherosclerosis signalling, IL‐12 signalling and production in macrophages, coagulation system and complement system (Table 3). The interactive relationship between proteins in the first canonical pathway is denoted in Figure 4. The whole information on the canonical pathway output is displayed in Table S3. It is noteworthy to mention that the top one network is intimately associated with lipid metabolism (Fig. 4).

**Table 3:** The top seven canonical pathways enriched by IPA. The 20 differentially expressed proteins were imported to IPA software, and the generated top seven pathways are shown

Top seven canonical pathways	−log(*P*‐value)	Molecules	Ratio
LXR/RXR Activation	1.47E01	9	7.44E‐02
Acute phase response signaling	1.34E01	9	5.36E‐02
FXR/RXR Activation	1.05E01	7	5.6E‐02
Atherosclerosis signaling	5.13E00	4	3.23E‐02
IL‐12 Signaling and production in macrophages	3.37E00	3	2.08E‐02
Coagulation system	3.22E00	2	5.71E‐02
Complement system	3.2E00	2	5.56E‐02

### Hierarchical clustering analysis

**Figure 5:**
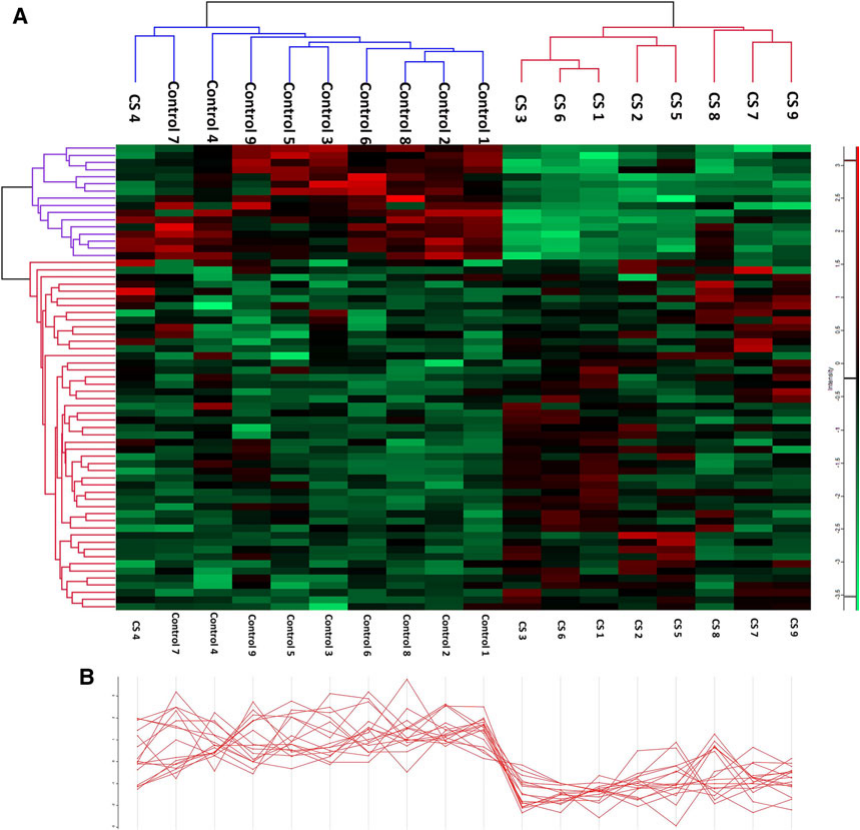
Hierarchical clustering analysis of 65 proteins with *q* values < 0.05. (**A**) Heat map representation of every protein in the congenital scoliosis (CS) and control samples. The proteins were separated into two clusters: cluster 61 (16 proteins) and cluster 62 (49 proteins). Each row represents an individual protein, and each column represents an individual sample. The dendrogram at the top suggests the similarity in protein expression profiles of the sample. (**B**) The expressional pattern of each protein in cluster 61 across every individual sample. Blue–red heat map values correspond to low–high protein expression levels.

We conducted the hierarchical clustering analysis on the basis of the expression values for the 65 proteins having *q* values < 0.05. The data were Z‐score transformed and were clustered in both the row and the column directions. The results showed that most of these samples were correctly classified into two groups corresponding to disease or normal control condition, apart from one sample (CS 4) that was incorrectly classified (Fig. 5A). This suggests that CS may have particular expression profiles, distinct from the normal state profiles. The 65 submitted proteins were separated into two clusters: cluster 61 (16 proteins) and cluster 62 (49 proteins) (Table S4). The corresponding expressional pattern in cluster 61 is delineated in Figure 5B.

## Discussion

In the present study, to elucidate the mechanisms of *TBX6*‐induced CS at the protein level, we employed a comparative proteomics strategy of iTRAQ to explore the differential protein expression profiles, GO terms, IPA network and pathway and hierarchical clustering between normal control and CS patients with both *TBX6* deletion and T‐C‐A haplotype. To the best of our knowledge, this investigation is the first proteomics research on CS, following our latest genetic revelation of *TBX6* in the onset of CS 1.

Our results indicated that 277 total proteins were expressed in both CS and normal controls. Furthermore, 65 proteins showed *q* values < 0.05 and 20 proteins were designated as differentially expressed proteins (*q* < 0.05, and ratio ≥ 1.42 or ≤0.70). We then conducted bioinformatics analyses on these proteins. Generally, the GO classification suggested that annotation with highest percentage in biological process, molecular function and cellular component was cellular process, structural molecule activity and extracellular region, respectively. IPA output showed that the most significant canonical pathway was the ‘LXR/RXR activation’ pathway. Hierarchical clustering analysis separated the 65 protein (*q* < 0.05) into two clusters (16 *versus* 49 proteins). Due to the significant expression modulations as well as potential biological and functional relevance of the differentially expressed proteins in the pathogenesis and aetiology of CS, we herein choose several of the proteins to discuss in the following section.

The most appealing finding to emerge from our study is that we, for the first time, hypothesize lipid metabolism to play vital roles in the pathogenesis of CS. Supporting evidence not only arises from proteomic revelation of APOC1 and LXR/RXR pathway in this study, as illustrated in following text, but also comes from other discoveries including the doctoral thesis of Dr. Sun from our team who conducted a preliminary proteomics analysis on CS by 2‐D gel electrophoresis in 2011 (http://d.g.wanfangdata.com.cn/Thesis_Y1985714.aspx). Part of the serum proteins he found, such as APOA1 and APOA4, also point to lipid metabolism. But his results have not been submitted to any journal for publishing so far. LXR encodes highly homologous transcription factors of nuclear receptors and forms functional heterodimers with RXR *via* LXR‐responsive element within the targeted regulators 13. The LXR/RXR functions as sensors of cholesterol homoeostasis by regulating genes that control sterol and fatty acid metabolism 14. Another supporting evidence is that in adolescent idiopathic scoliosis, which is another type of scoliosis and may have overlapping pathogenesis with CS 15, 16, lipid metabolism was identified to be the most remarkable metabolic changes in the serum 17. Taken together, our results and other evidence strongly point to disrupted lipid metabolism in CS. More generalized studies with larger sample sizes should be conducted to investigate whether lipid metabolism is associated with CS.

Sex hormone‐binding globulin (SHBG) is a homodimeric glycoprotein, the most prominent feature of which is to bind sex steroids with equally high affinity as well as specificity. It is synthesized in the liver, and its molecular weight is approximately 90 kD 18. Each dimer of the SHBG, consisting of 373 amino acids, exhibits a tertiary structure of carboxy‐terminal laminin G‐like domain (LGLD), which is joined to an amino‐terminal LGLD by a linker region 19. SHBG has been reported to be implicated in various physiological disturbance states and diseases. In polycystic ovary syndrome (PCOS), Wassell *et al*. 20 found that PCOS patients exhibiting a positive response to metformin treatment showed a significantly lower pre‐treatment SHBG levels, implying the SHBG may be a predictor of response to pharmacological treatment for PCOS. In thyroid disorders, Hampl *et al*. 21 observed that low SHBG levels in patients treated with total thyroidectomy because of thyroid cancer were significantly elevated to physiological values after reaching normal thyroid function. In pituitary diseases, hyperprolactinemia was associated with decreased SHBG concentrations, and SHBG levels returned to normal limits following bromocriptine administration 22. In metabolic syndrome (METS) and type 2 diabetes mellitus (T2DM), SHBG levels have been demonstrated to have an inverse association with an increased risk for the development of METS and T2DM 23, 24, 25, suggesting that SHBG is likely to represent a sensitive and early biomarker for metabolic disturbances. The related molecular mechanism explaining the observational studies may be partially attributed to that monosaccharide‐induced hepatic *de novo* lipogenesis can reduce hepatic HNF‐4α levels and subsequently *SHBG* gene expression. In liver disorders, SHBG was significantly increased in patients with cirrhosis, a result and final path of various liver disorders 26. The elevation of SHBG in cirrhosis was less pronounced with progression to the decompensated state. As liver cirrhosis is inclined to develop into hepatocellular carcinoma (HCC), SHBG might constitute a potential risk marker for early detection of HCC 26. In the realm of orthopaedic researches, SHBG was found abated in rheumatoid arthritis patients than control volunteers, in both pre‐menopausal and postmenopausal status 27. SHBG was also associated with bone loss, vertebral fractures and kyphosis 28. Interestingly, Chilean scholars, Albala *et al*. 29, evaluated the bone mineral density and the SHBG in obese and non‐obese postmenopausal women, and concluded that obesity exerts protection against osteoporosis *via* decreased SHBG. Furthermore, another publication suggested that high serum SHBG levels have an increased risk of fractures in older Swedish men 30. Low bone mineral density has been observed in CS patients 31, and the distribution of bone mineral density in CS mice was highest in the curve apex 32. Our study is the first to account for SHBG protein as differentially expressed protein in CS patients. We believe the finding of SHBG may be helpful in elaborating the low bone mineral density in CS patients when taking into account the reported role of SHBG in osteoporosis.

Apolipoprotein C‐I (APOC1), with a 6.6 kD molecular mass, is one constituent of chylomicrons, high‐density lipoprotein (HDL) and triglyceride‐rich lipoproteins, which can blunt the metabolism of triglyceride‐rich lipoproteins 33. APOC1 functions as an inhibitor of lipoprotein binding to the low‐density lipoprotein (LDL) receptor and very low‐density lipoprotein (VLDL) receptor. APOC1 is also a major inhibitor of cholesterol ester transfer protein and is able to affect fatty acid uptake in a direct manner 34. High expression of apolipoproteins in embryogenesis would be helpful for transporting material, such as lipid. Therefore, it is reasonable to speculate APOC1, the smallest member of apolipoprotein family, may have an important role in embryogenesis, in addition to the well‐documented expression of other lipoproteins (APOE and APOA1) in embryonic development 35 that, potentially, may be associated with bone forming. One publication confirmed our ratiocination. Wang and his colleagues 36 observed in zebrafish that apoc1 gene is expressed in gradient along the ventral–dorsal axis with the highest expression on the ventral side, and apoc1 gene expression occurs in tail paraxial mesoderm that originates from the blastoderm margin in somitogenesis. They also revealed that apoc1 gene expression can be activated by bone morphogenetic proteins (BMPs) signalling, and this effect can be dampened by retinoic acid (RA) signalling suppression. This serves a new cynosure because RA and its receptors are demonstrated to be expressed in the paraxial mesoderm and are critical for regulating key genes in the somitogenesis clock and the HOX gene induction 37. RA inhibits the ability of the paraxial mesoderm to respond to asymmetric signalling downstream of the left‐side determinant Nodal, which could cause asymmetry of somitogenesis 38. In addition, BMP signalling has been shown to be involved in the development of the initial vertebrate body and somite 39, 40 and BMP inhibitor, dorsomorphin, produces split‐body phenotypes including bilaterally split somites 41. Considering the roles of BMP and RA in somitogenesis and their demonstrated downstream effects on APOC1 protein 36, we can infer that the down‐regulated expression of APOC1 protein in our study and the occurrence of CS are two concurrent phenotypes caused, at least in part, by dysregulation of some common upstream signallings such as RA and BMP. Furthermore, apoc1 gene expression in murine macrophages can be up‐regulated by ligands of LXR/RXR pathway 42, which is in agreement with the appearance of LXR/RXR pathway as the most remarkable canonical pathway of IPA output in our study. This further strengthens the link between APOC1 and CS. As LXR/RXR pathway is the key regulator for lipid and cholesterol metabolism 14, it is reasonable to generalize a step further from APOC1 and LXR/RXR to lipid metabolism – it is lipid metabolism that may share a common molecular background with CS under partial contributions from RA and BMPs. Given the previously demonstrated aetiological documentation of CS, we can even propose a more radical supposition that lipid metabolism may have interactions with the known mechanisms of CS, and future investigations on how the lipid metabolism is involved in the onset of CS, especially from an embryological point of view, may shed new light on the early prevention and treatment of CS.

Complement C4‐A (CO4A) is a component of the complement system and is indispensable for its classical activation pathway 43. CO4A is derived from the proteolytic degradation of complement factor 4 (C4). CO4A acts in the form of a single chain precursor, and then is cleaved into a trimer before secretion 44. Anaphylatoxin C4a, the cleaved product of alpha chain of C4, is a mediator of local inflammatory response. The gene encoding CO4A is localized at the major histocompatibility complex (MHC) class III region. Recent studies implicated that CO4A was related to neurological disorders. In schizophrenia, the level of C4 protein was observed to be significantly increased in patients' sera 45, 46, and CO4A RNA expression was significantly higher in the brain tissue 47. Besides, *C4A* gene structural diversity can in part serve as a bridge between MHC variation and schizophrenia 47. It is known that C4 protein lies in neuronal synapses, dendrites, axons and cell bodies. Thus, the schizophrenic phenotype can be attributed to the potential role of C4 protein in synapse remodelling and pruning during the neurodevelopmental process 48. In our study, the level of CO4A protein was significantly elevated in CS patients, all of which carry a 16p11.2 deletion. In fact, 16p11.2 deletion has demonstrated to be a likely cause for developmental delay, mental retardation and autism spectrum disorder 49. However, the detailed neurodevelopmental records of the patients in this study were not available for us to determine whether they had neurological disorders or not. Further investigations are needed to clarify this point. As CS and neurological disorder (such as schizophrenia) are two well‐demonstrated phenotypes caused by 16p11.2 deletion, one phenotype may, to some extent, be indicative of the other phenotype. Hence, we propose that CO4A, and thus neurological disorder (which has to do with CO4A) may be coincidental with some CS cases that have 16p11.2 deletion. In line with our reasoning pattern in the hypothesis, previous publications revealed that two other phenotypes of 16p11.2 deletion, obesity and low cognitive functioning, showed remarkable interrelations with each other: people who have learning disabilities have a greater chance of being obese 50, and the obese people in turn exhibit a substantially decreased cognitive ability 51. However, the altered level of CO4A in our study might be caused by 16p11.2 deletion, but not CS. Whether CO4A and CS can manifest simultaneously depends on the coverage of deletion.

Transgelin‐2 is a homolog of the protein transgelin, a 22 kD actin‐binding protein of the calponin family. Transgelin was first identified in 1987 in smooth muscle tissues of birds and therefore named as smooth muscle 22 (SM22) 52. Transgelin is widely expressed in vascular and visceral smooth muscles and is one of the earliest markers of smooth muscle differentiation 53. Transgelin‐2 has been suggested to be a tumour suppressor demonstrated by its discovery as differentially expressed protein in several proteomics studies. Through the 2‐D gel electrophoresis, transgelin‐2 was identified by MS as one of the differentially expressed proteins in lung adenocarcinoma 54. Validation by immunohistochemistry revealed the exclusive expression of transgelin and transgelin‐2 in the stromal tissue compartment and neoplastic glandular compartment, respectively, suggesting its important role in active stromal remodelling of invasive carcinomas. Similarly, in a highly metastatic variant of human breast cancer cell, transgelin‐2 was found to be the only down‐regulated protein, and verification experiments implied a significant association between lymph node metastasis and expression of transgelin‐2 55. In antineoplastic drug‐resistant breast cancer cell line, transgelin‐2 was reported to be a mediator for drug resistance by up‐regulating the adenosine triphosphate‐binding cassette transporter proteins 56. In colorectal cancer, transgelin‐2 was significantly up‐regulated, which was also discovered by quantitative proteome analysis. Further validation indicated transgelin‐2 was tissue‐specific in colorectal cancer cells, and overexpression of transgelin‐2 was associated with tumour prognostic factors 57. Apart from its identification in cancer cells, transgelin‐2 was also significantly differentially expressed in proteome of other diseases, including rheumatoid arthritis and meniscal injury. Transgelin‐2 was selected as one of the candidate markers for rheumatoid arthritis due to its elevated expression in the synovial fluid of erosive rheumatoid arthritis in comparison with non‐erosive rheumatoid arthritis 58. In injured menisci, proteomic analysis suggested transgelin‐2 was one of the interest proteins after evaluating the expressional trends between case and control groups 59. In spite of its discovery and potential biological value in many diseases, transgelin‐2 has never been reported in the threshold of scoliosis research. Our study for the first time reports the involvement of transgelin‐2 as serum differentially expressed protein in CS. As such, transgelin‐2 may have been suggested as a candidate marker for CS possibly indicating potential co‐existing smooth muscle deformity with the malformation of spine. However, more observational investigations, especially on abnormal embryogenesis, are necessary to confirm the hypothesis.

In addition to the above‐mentioned proteins, other significantly differentially expressed proteins, including serum amyloid A‐1 protein, serum amyloid A‐2 protein, leucine‐rich α‐2‐glycoprotein, creatine kinase M‐type and α‐1‐acid glycoprotein 1, are also worth our concern. Serum amyloid A proteins belong to the apolipoproteins family and are acute phase proteins during inflammatory response. Serum amyloid A‐1 and A‐2, two isoforms of serum amyloid A, are produced by hepatocyte and released into blood up to a 1000‐fold during inflammation 60. Leucine‐rich α‐2‐glycoprotein is a potential diagnostic marker for inflammation‐associated diseases for its augmented expression in ulcerative colitis 61, 62, asthma 63, acute appendicitis 64 and rheumatoid arthritis 65. Creatine kinase M‐type, a subtype of creatine kinase, reversibly catalyses the conversion of creatine into phosphocreatine and adenosine diphosphate (ADP) by facilitating the transfer of adenosine triphosphate (ATP) 66. Because it is mainly expressed in sarcomeric muscles such as skeletal and cardiac muscles, creatine kinase M‐type is a potential diagnostic marker for myocardial infarction 67, rhabdomyolysis 68 and myositis 69. α‐1‐acid glycoprotein 1 is an acute phase protein involved in immune system modulation, and elevated serum α‐1‐acid glycoprotein 1 is seen in systemic inflammatory response or infection 70. Although these proteins are firstly identified in the proteome of CS, future researches on the possible biological merits of the observed proteins are still necessary for the aetiological elucidation of CS.

In summary, we for the first time depicted the differential protein expression profiles of CS in serum. These previously unidentified proteins may offer invaluable clue for not only the aetiological mechanism but also the onset and development of CS. The application of these proteins may serve effective diagnostic tools for early detection of CS. The underlying interactions between the differential proteins and CS need further investigation.

## **Conflict of interest**

The authors confirm that there is no conflict of interests.

## Supporting information


**Table S1** List of total proteins identified in sera from CS and control samples.

**Table S2** Network analysis results generated by IPA software.

**Table S3** Ingenuity canonical pathways assigned by IPA software.

**Table S4** The separated proteins in cluster 61 and cluster 62 by HCA.
