# Supplementary material for: Comparative analysis of serum proteome in congenital scoliosis patients with TBX6 haploinsufficiency – a first report pointing to lipid metabolism
Source: J Cell Mol Med. 2017 Sep 25;22(1):533–45. doi: 10.1111/jcmm.13341 (PMC5742745; doi:10.1111/jcmm.13341)
Supplement: Supplementary file 1 — Table S1 List of total proteins identified in sera from CS and control samples. [file JCMM-22-533-s001.doc]

# Comparative analysis of serum proteome in congenital scoliosis patients with *TBX6* haploinsufficiency- a first report pointing to lipid metabolism

Qiankun Zhu^1﹟^, Nan Wu^1, 2, 3﹟^, Gang Liu^1, 2, 3^, Yangzhong Zhou^4^, Sen Liu^1^, Jun Chen^5^, Jiaqi Liu^1^, Yuzhi Zuo^1^, Zhenlei Liu^7^, Weisheng Chen^1^, Yixin Chen^1^, Jia Chen^1^, Mao Lin^1^, Yanxue Zhao^1^, Yang Yang^1^, Shensgru Wang^1^, Xu Yang^1^, Yufen Ma^1^, Jian Wang^8^, Xiaoli Chen^9^, Jianguo Zhang^1^, Jianxiong Shen^1^, Zhihong Wu ^2,3,6*^, Guixing Qiu^1, 2, 3*^

1. Department of Orthopedic Surgery, Peking Union Medical College Hospital, Peking Union Medical College and Chinese Academy of Medical Sciences, Beijing, China
2. Beijing Key Laboratory for Genetic Research of Skeletal Deformity, China
3. Research Center of Orthopedics/Rare Disease, Chinese Academy of Medical Sciences, Beijing, China
4. Tsinghua University Medical School, Beijing, China
5. Department of Pathology, Beijing Ditan Hospital, Capital Medical University, Beijing, China
6. Department of Central Laboratory, Peking Union Medical College Hospital, Peking Union Medical College and Chinese Academy of Medical Sciences, Beijing, China
7. Department of Neurosurgery, Xuanwu Hospital, Capital Medical University, Beijing, China
8. Department of Medical Genetics, Molecular Diagnostic Laboratory, Shanghai Children's Medical Center, Shanghai Jiaotong University School of Medicine, Shanghai, China
9. Department of Medical Genetics, Beijing Municipal Key Laboratory of Child Development and Nutriomics, Capital Institute of Pediatrics, Beijing, China

*Correspondence to: Dr. Guixing Qiu, Department of Orthopedic Surgery, Peking Union Medical College Hospital; Beijing Key Laboratory for Genetic Research of Skeletal Deformity; Research Center of Orthopedics/Rare Disease, Chinese Academy of Medical Sciences, No.1 Shuaifuyuan, Beijing, China. Tel: +8601069152809, E-mail: [qiuguixingpumch@126.com](mailto:qiuguixingpumch@126.com). Zhihong Wu, Beijing Key Laboratory for Genetic Research of Skeletal Deformity, Research Center of Orthopedics/Rare Disease, Department of Central Laboratory, Peking Union Medical College Hospital, Peking Union Medical College and Chinese Academy of Medical Sciences, No.1 Shuaifuyuan, Beijing, China. Tel: +8601069154259, E-mail: [orthoscience@126.com](mailto:orthoscience@126.com).

Guixing Qiu and Zhihong Wu are co-corresponding authors.

The authors declare that there is no conflict of interest.

﹟These authors contributed equally to this work.

| Accession number | Protein name | CS mean | CS SD | Control mean | Control SD | Ratio (CS/control) | Up-or down-regulation | -Log P value | q value | Differentially expressed proteins |
| --- | --- | --- | --- | --- | --- | --- | --- | --- | --- | --- |
| CO9_HUMAN | Complement component C9 OS=Homo sapiens GN=C9 PE=1 SV=2 | 17.432 | 0.220 | 16.912 | 0.165 | 1.434 | UP | 4.463 | 0.000 | + |
| TAGL2_HUMAN | Transgelin-2 OS=Homo sapiens GN=TAGLN2 PE=1 SV=3 | 15.599 | 0.287 | 14.964 | 0.233 | 1.552 | UP | 4.014 | 0.000 | + |
| CRP_HUMAN | C-reactive protein OS=Homo sapiens GN=CRP PE=1 SV=1 | 16.620 | 0.801 | 15.297 | 0.210 | 2.502 | UP | 3.702 | 0.002 | + |
| LBP_HUMAN | Lipopolysaccharide-binding protein OS=Homo sapiens GN=LBP PE=1 SV=3 | 16.160 | 0.407 | 15.331 | 0.343 | 1.776 | UP | 3.593 | 0.003 | + |
| SHBG_HUMAN | Sex hormone-binding globulin OS=Homo sapiens GN=SHBG PE=1 SV=2 | 16.518 | 0.254 | 17.181 | 0.335 | 0.631 | Down | 3.643 | 0.003 | + |
| VWF_HUMAN | von Willebrand factor OS=Homo sapiens GN=VWF PE=1 SV=4 | 16.863 | 0.303 | 16.356 | 0.178 | 1.422 | UP | 3.293 | 0.003 | + |
| MMP9_HUMAN | Matrix metalloproteinase-9 OS=Homo sapiens GN=MMP9 PE=1 SV=3 | 15.666 | 0.313 | 15.151 | 0.216 | 1.428 | UP | 3.041 | 0.005 | + |
| A1AG1_HUMAN | Alpha-1-acid glycoprotein 1 OS=Homo sapiens GN=ORM1 PE=1 SV=1 | 17.388 | 0.269 | 16.872 | 0.289 | 1.430 | UP | 2.914 | 0.008 | + |
| SAA2_HUMAN | Serum amyloid A-2 protein OS=Homo sapiens GN=SAA2 PE=1 SV=1 | 17.382 | 0.453 | 16.777 | 0.169 | 1.522 | UP | 2.766 | 0.010 | + |
| K1C10_HUMAN | Keratin, type I cytoskeletal 10 OS=Homo sapiens GN=KRT10 PE=1 SV=6 | 16.266 | 0.080 | 16.844 | 0.507 | 0.669 | Down | 2.422 | 0.016 | + |
| A2GL_HUMAN | Leucine-rich alpha-2-glycoprotein OS=Homo sapiens GN=LRG1 PE=1 SV=2 | 16.537 | 0.528 | 15.868 | 0.266 | 1.590 | UP | 2.430 | 0.017 | + |
| CO4A_HUMAN | Complement C4-A OS=Homo sapiens GN=C4A PE=1 SV=2 | 17.520 | 0.278 | 16.919 | 0.468 | 1.517 | UP | 2.357 | 0.018 | + |
| K2C1_HUMAN | Keratin, type II cytoskeletal 1 OS=Homo sapiens GN=KRT1 PE=1 SV=6 | 16.021 | 0.171 | 16.823 | 0.721 | 0.573 | Down | 2.296 | 0.020 | + |
| HBG2_HUMAN | Hemoglobin subunit gamma-2 OS=Homo sapiens GN=HBG2 PE=1 SV=2 | 15.672 | 0.503 | 16.409 | 0.482 | 0.600 | Down | 2.228 | 0.023 | + |
| KCRM_HUMAN | Creatine kinase M-type OS=Homo sapiens GN=CKM PE=1 SV=2 | 15.127 | 0.469 | 14.556 | 0.279 | 1.486 | UP | 2.198 | 0.024 | + |
| APOC1_HUMAN | Apolipoprotein C-I OS=Homo sapiens GN=APOC1 PE=1 SV=1 | 17.607 | 0.543 | 18.237 | 0.319 | 0.646 | Down | 2.074 | 0.029 | + |
| K22E_HUMAN | Keratin, type II cytoskeletal 2 epidermal OS=Homo sapiens GN=KRT2 PE=1 SV=2 | 14.629 | 0.233 | 15.213 | 0.540 | 0.667 | Down | 2.055 | 0.030 | + |
| SAA1_HUMAN | Serum amyloid A-1 protein OS=Homo sapiens GN=SAA1 PE=1 SV=1 | 16.743 | 0.756 | 15.982 | 0.225 | 1.695 | UP | 1.977 | 0.032 | + |
| IGHG1_HUMAN | Ig gamma-1 chain C region OS=Homo sapiens GN=IGHG1 PE=1 SV=1 | 16.583 | 0.548 | 17.162 | 0.350 | 0.669 | Down | 1.777 | 0.045 | + |
| A1AT_HUMAN | Alpha-1-antitrypsin OS=Homo sapiens GN=SERPINA1 PE=1 SV=3 | 17.018 | 0.559 | 16.487 | 0.235 | 1.445 | UP | 1.737 | 0.048 | + |
| FA9_HUMAN | Coagulation factor IX OS=Homo sapiens GN=F9 PE=1 SV=2 | 17.601 | 0.128 | 17.356 | 0.063 | 1.186 | UP | 4.030 | 0.000 | - |
| PROF1_HUMAN | Profilin-1 OS=Homo sapiens GN=PFN1 PE=1 SV=2 | 15.152 | 0.132 | 14.792 | 0.116 | 1.283 | UP | 4.853 | 0.000 | - |
| PEDF_HUMAN | Pigment epithelium-derived factor OS=Homo sapiens GN=SERPINF1 PE=1 SV=4 | 16.990 | 0.150 | 16.699 | 0.088 | 1.224 | UP | 3.910 | 0.001 | - |
| CO6_HUMAN | Complement component C6 OS=Homo sapiens GN=C6 PE=1 SV=3 | 17.628 | 0.170 | 17.342 | 0.075 | 1.219 | UP | 3.535 | 0.003 | - |
| CO8G_HUMAN | Complement component C8 gamma chain OS=Homo sapiens GN=C8G PE=1 SV=3 | 17.026 | 0.111 | 16.824 | 0.083 | 1.150 | UP | 3.305 | 0.004 | - |
| VTNC_HUMAN | Vitronectin OS=Homo sapiens GN=VTN PE=1 SV=1 | 17.277 | 0.170 | 16.998 | 0.098 | 1.213 | UP | 3.231 | 0.004 | - |
| CO3_HUMAN | Complement C3 OS=Homo sapiens GN=C3 PE=1 SV=2 | 17.002 | 0.133 | 16.766 | 0.102 | 1.178 | UP | 3.199 | 0.004 | - |
| CNDP1_HUMAN | Beta-Ala-His dipeptidase OS=Homo sapiens GN=CNDP1 PE=1 SV=4 | 17.124 | 0.290 | 16.629 | 0.174 | 1.410 | UP | 3.346 | 0.004 | - |
| TSP1_HUMAN | Thrombospondin-1 OS=Homo sapiens GN=THBS1 PE=1 SV=2 | 17.001 | 0.158 | 16.644 | 0.185 | 1.280 | UP | 3.349 | 0.004 | - |
| GP1BA_HUMAN | Platelet glycoprotein Ib alpha chain OS=Homo sapiens GN=GP1BA PE=1 SV=2 | 16.406 | 0.133 | 16.112 | 0.149 | 1.225 | UP | 3.356 | 0.005 | - |
| A1AG2_HUMAN | Alpha-1-acid glycoprotein 2 OS=Homo sapiens GN=ORM2 PE=1 SV=2 | 17.596 | 0.225 | 17.219 | 0.161 | 1.298 | UP | 3.066 | 0.005 | - |
| ACTB_HUMAN (+1) | Actin, cytoplasmic 1 OS=Homo sapiens GN=ACTB PE=1 SV=1 | 16.327 | 0.270 | 15.914 | 0.142 | 1.331 | UP | 3.041 | 0.006 | - |
| APOA4_HUMAN | Apolipoprotein A-IV OS=Homo sapiens GN=APOA4 PE=1 SV=3 | 17.769 | 0.175 | 18.122 | 0.201 | 0.783 | Down | 2.968 | 0.006 | - |
| C4BPB_HUMAN | C4BPB_HUMAN | 16.938 | 0.172 | 16.666 | 0.128 | 1.208 | UP | 2.818 | 0.010 | - |
| NRP1_HUMAN | Neuropilin-1 OS=Homo sapiens GN=NRP1 PE=1 SV=3 | 17.114 | 0.140 | 16.858 | 0.145 | 1.195 | UP | 2.827 | 0.010 | - |
| FA10_HUMAN | Coagulation factor X OS=Homo sapiens GN=F10 PE=1 SV=2 | 17.612 | 0.134 | 17.403 | 0.114 | 1.156 | UP | 2.589 | 0.013 | - |
| OAF_HUMAN | Out at first protein homolog OS=Homo sapiens GN=OAF PE=2 SV=1 | 15.448 | 0.282 | 15.090 | 0.119 | 1.281 | UP | 2.538 | 0.013 | - |
| CO8A_HUMAN | Complement component C8 alpha chain OS=Homo sapiens GN=C8A PE=1 SV=2 | 16.668 | 0.102 | 16.521 | 0.071 | 1.107 | UP | 2.554 | 0.013 | - |
| APOB_HUMAN | Apolipoprotein B-100 OS=Homo sapiens GN=APOB PE=1 SV=2 | 16.688 | 0.307 | 17.160 | 0.252 | 0.721 | Down | 2.590 | 0.014 | - |
| LG3BP_HUMAN | Galectin-3-binding protein OS=Homo sapiens GN=LGALS3BP PE=1 SV=1 | 15.999 | 0.196 | 15.666 | 0.217 | 1.260 | UP | 2.456 | 0.016 | - |
| ZPI_HUMAN | Protein Z-dependent protease inhibitor OS=Homo sapiens GN=SERPINA10 PE=1 SV=1 | 16.883 | 0.109 | 16.668 | 0.158 | 1.161 | UP | 2.406 | 0.017 | - |
| CFAI_HUMAN | Complement factor I OS=Homo sapiens GN=CFI PE=1 SV=2 | 16.843 | 0.249 | 16.512 | 0.162 | 1.258 | UP | 2.387 | 0.017 | - |
| TLN1_HUMAN | TLN1_HUMAN | 14.282 | 0.188 | 13.980 | 0.216 | 1.233 | UP | 2.222 | 0.023 | - |
| APOH_HUMAN | Beta-2-glycoprotein 1 OS=Homo sapiens GN=APOH PE=1 SV=3 | 17.402 | 0.239 | 17.100 | 0.164 | 1.233 | UP | 2.189 | 0.025 | - |
| PLF4_HUMAN | Platelet factor 4 OS=Homo sapiens GN=PF4 PE=1 SV=2 | 16.426 | 0.174 | 16.213 | 0.110 | 1.158 | UP | 2.151 | 0.027 | - |
| FHR3_HUMAN | Complement factor H-related protein 3 OS=Homo sapiens GN=CFHR3 PE=1 SV=2 | 16.959 | 0.327 | 16.506 | 0.299 | 1.369 | UP | 2.138 | 0.027 | - |
| C4BPA_HUMAN | C4b-binding protein alpha chain OS=Homo sapiens GN=C4BPA PE=1 SV=2 | 17.500 | 0.212 | 17.264 | 0.097 | 1.177 | UP | 2.101 | 0.029 | - |
| HV303_HUMAN | Ig heavy chain V-III region 23 OS=Homo sapiens GN=IGHV3-23 PE=1 SV=2 | 17.053 | 0.355 | 17.481 | 0.245 | 0.743 | Down | 2.049 | 0.030 | - |
| LV301_HUMAN | Ig lambda chain V-III region SH OS=Homo sapiens PE=1 SV=1 | 16.009 | 0.396 | 16.467 | 0.239 | 0.728 | Down | 2.042 | 0.030 | - |
| C1R_HUMAN | Complement C1r subcomponent OS=Homo sapiens GN=C1R PE=1 SV=2 | 16.943 | 0.207 | 16.712 | 0.116 | 1.174 | UP | 2.003 | 0.031 | - |
| PROC_HUMAN | Vitamin K-dependent protein C OS=Homo sapiens GN=PROC PE=1 SV=1 | 16.352 | 0.121 | 16.211 | 0.082 | 1.103 | UP | 1.977 | 0.031 | - |
| AMBP_HUMAN | Protein AMBP OS=Homo sapiens GN=AMBP PE=1 SV=1 | 16.590 | 0.234 | 16.332 | 0.119 | 1.196 | UP | 2.017 | 0.032 | - |
| APOA1_HUMAN | Apolipoprotein A-I OS=Homo sapiens GN=APOA1 PE=1 SV=1 | 16.983 | 0.314 | 17.334 | 0.180 | 0.784 | Down | 1.991 | 0.032 | - |
| KV402_HUMAN | Ig kappa chain V-IV region Len OS=Homo sapiens PE=1 SV=2 | 15.911 | 0.290 | 16.257 | 0.199 | 0.787 | Down | 2.022 | 0.032 | - |
| F13B_HUMAN | Coagulation factor XIII B chain OS=Homo sapiens GN=F13B PE=1 SV=3 | 16.124 | 0.189 | 15.890 | 0.148 | 1.176 | UP | 2.005 | 0.032 | - |
| IGHM_HUMAN | Ig mu chain C region OS=Homo sapiens GN=IGHM PE=1 SV=3 | 16.450 | 0.250 | 16.830 | 0.320 | 0.768 | Down | 1.899 | 0.035 | - |
| FBLN1_HUMAN | Fibulin-1 OS=Homo sapiens GN=FBLN1 PE=1 SV=4 | 17.558 | 0.325 | 17.211 | 0.171 | 1.272 | UP | 1.921 | 0.035 | - |
| C1S_HUMAN | Complement C1s subcomponent OS=Homo sapiens GN=C1S PE=1 SV=1 | 16.788 | 0.198 | 16.584 | 0.086 | 1.151 | UP | 1.913 | 0.035 | - |
| MA1A1_HUMAN | Mannosyl-oligosaccharide 1,2-alpha-mannosidase IA OS=Homo sapiens GN=MAN1A1 PE=1 SV=3 | 15.268 | 0.298 | 14.924 | 0.213 | 1.269 | UP | 1.901 | 0.035 | - |
| HV102_HUMAN | Ig heavy chain V-I region HG3 OS=Homo sapiens PE=3 SV=1 | 16.204 | 0.279 | 16.627 | 0.348 | 0.746 | Down | 1.925 | 0.035 | - |
| IGHA1_HUMAN | Ig alpha-1 chain C region OS=Homo sapiens GN=IGHA1 PE=1 SV=2 | 17.209 | 0.170 | 16.951 | 0.227 | 1.196 | UP | 1.828 | 0.043 | - |
| CD14_HUMAN | Monocyte differentiation antigen CD14 OS=Homo sapiens GN=CD14 PE=1 SV=2 | 16.810 | 0.272 | 16.553 | 0.089 | 1.195 | UP | 1.793 | 0.045 | - |
| HABP2_HUMAN | Hyaluronan-binding protein 2 OS=Homo sapiens GN=HABP2 PE=1 SV=1 | 17.482 | 0.241 | 17.228 | 0.158 | 1.193 | UP | 1.760 | 0.046 | - |
| PLMN_HUMAN | Plasminogen OS=Homo sapiens GN=PLG PE=1 SV=2 | 17.824 | 0.192 | 17.638 | 0.083 | 1.138 | UP | 1.777 | 0.046 | - |
| LV106_HUMAN | Ig lambda chain V-I region WAH OS=Homo sapiens PE=1 SV=1 | 17.553 | 0.321 | 17.917 | 0.265 | 0.777 | Down | 1.733 | 0.048 | - |
| CFAH_HUMAN | Complement factor H OS=Homo sapiens GN=CFH PE=1 SV=4 | 17.241 | 0.157 | 17.069 | 0.125 | 1.127 | UP | 1.694 | 0.051 | - |
| 1433Z_HUMAN | 14-3-3 protein zeta/delta OS=Homo sapiens GN=YWHAZ PE=1 SV=1 | 16.232 | 0.291 | 15.909 | 0.247 | 1.251 | UP | 1.662 | 0.052 | - |
| FETUA_HUMAN | Alpha-2-HS-glycoprotein OS=Homo sapiens GN=AHSG PE=1 SV=1 | 17.387 | 0.145 | 17.206 | 0.156 | 1.134 | UP | 1.668 | 0.052 | - |
| AACT_HUMAN | Alpha-1-antichymotrypsin OS=Homo sapiens GN=SERPINA3 PE=1 SV=2 | 16.950 | 0.442 | 16.548 | 0.160 | 1.322 | UP | 1.683 | 0.052 | - |
| HV209_HUMAN | Ig heavy chain V-II region ARH-77 OS=Homo sapiens PE=4 SV=1 | 17.360 | 0.314 | 17.722 | 0.287 | 0.778 | Down | 1.673 | 0.053 | - |
| CBG_HUMAN | Corticosteroid-binding globulin OS=Homo sapiens GN=SERPINA6 PE=1 SV=1 | 17.369 | 0.227 | 17.600 | 0.149 | 0.852 | Down | 1.669 | 0.053 | - |
| LV001_HUMAN | Ig lambda chain V region 4A OS=Homo sapiens PE=4 SV=1 | 17.830 | 0.435 | 18.286 | 0.323 | 0.729 | Down | 1.643 | 0.055 | - |
| KV305_HUMAN | Ig kappa chain V-III region WOL OS=Homo sapiens PE=1 SV=1 | 16.134 | 0.394 | 16.530 | 0.280 | 0.760 | Down | 1.587 | 0.063 | - |
| K1C9_HUMAN | Keratin, type I cytoskeletal 9 OS=Homo sapiens GN=KRT9 PE=1 SV=3 | 16.631 | 0.240 | 17.342 | 0.841 | 0.611 | Down | 1.573 | 0.064 | - |
| PHLD_HUMAN | Phosphatidylinositol-glycan-specific phospholipase D OS=Homo sapiens GN=GPLD1 PE=1 SV=3 | 17.053 | 0.153 | 16.906 | 0.100 | 1.108 | UP | 1.554 | 0.065 | - |
| HV304_HUMAN | Ig heavy chain V-III region TIL OS=Homo sapiens PE=1 SV=1 | 15.641 | 0.390 | 15.973 | 0.132 | 0.794 | Down | 1.558 | 0.065 | - |
| KV106_HUMAN | Ig kappa chain V-I region EU OS=Homo sapiens PE=1 SV=1 | 16.673 | 0.376 | 17.020 | 0.207 | 0.786 | Down | 1.559 | 0.065 | - |
| IGHA2_HUMAN | Ig alpha-2 chain C region OS=Homo sapiens GN=IGHA2 PE=1 SV=3 | 15.527 | 0.244 | 15.231 | 0.279 | 1.227 | UP | 1.532 | 0.068 | - |
| HV308_HUMAN | Ig heavy chain V-III region GA OS=Homo sapiens PE=1 SV=1 | 17.993 | 0.329 | 18.319 | 0.246 | 0.798 | Down | 1.520 | 0.069 | - |
| THRB_HUMAN | Prothrombin OS=Homo sapiens GN=F2 PE=1 SV=2 | 17.109 | 0.132 | 16.974 | 0.106 | 1.098 | UP | 1.520 | 0.070 | - |
| A1BG_HUMAN | Alpha-1B-glycoprotein OS=Homo sapiens GN=A1BG PE=1 SV=4 | 17.577 | 0.108 | 17.442 | 0.132 | 1.098 | UP | 1.504 | 0.071 | - |
| LAC2_HUMAN | Ig lambda-2 chain C regions OS=Homo sapiens GN=IGLC2 PE=1 SV=1 | 17.143 | 0.498 | 17.601 | 0.328 | 0.728 | Down | 1.455 | 0.082 | - |
| BTD_HUMAN | Biotinidase OS=Homo sapiens GN=BTD PE=1 SV=2 | 16.648 | 0.142 | 16.494 | 0.144 | 1.112 | UP | 1.426 | 0.087 | - |
| ANGT_HUMAN | Angiotensinogen OS=Homo sapiens GN=AGT PE=1 SV=1 | 17.152 | 0.176 | 16.988 | 0.130 | 1.121 | UP | 1.410 | 0.087 | - |
| A2MG_HUMAN | Alpha-2-macroglobulin OS=Homo sapiens GN=A2M PE=1 SV=3 | 16.971 | 0.306 | 17.211 | 0.091 | 0.847 | Down | 1.413 | 0.088 | - |
| CATD_HUMAN | Cathepsin D OS=Homo sapiens GN=CTSD PE=1 SV=1 | 16.177 | 0.191 | 15.996 | 0.152 | 1.134 | UP | 1.389 | 0.091 | - |
| CBPB2_HUMAN | CBPB2_HUMAN | 17.710 | 0.216 | 17.524 | 0.128 | 1.137 | UP | 1.382 | 0.092 | - |
| PRG4_HUMAN | Proteoglycan 4 OS=Homo sapiens GN=PRG4 PE=1 SV=2 | 16.880 | 0.283 | 16.643 | 0.163 | 1.178 | UP | 1.343 | 0.100 | - |
| KV101_HUMAN | Ig kappa chain V-I region AG OS=Homo sapiens PE=1 SV=1 | 16.197 | 0.478 | 16.616 | 0.327 | 0.748 | Down | 1.343 | 0.101 | - |
| IGLL5_HUMAN | Immunoglobulin lambda-like polypeptide 5 OS=Homo sapiens GN=IGLL5 PE=2 SV=2 | 17.368 | 0.263 | 17.679 | 0.344 | 0.806 | Down | 1.331 | 0.103 | - |
| HV306_HUMAN | Ig heavy chain V-III region BUT OS=Homo sapiens PE=1 SV=1 | 16.656 | 0.280 | 16.899 | 0.193 | 0.845 | Down | 1.324 | 0.103 | - |
| KV204_HUMAN | Ig kappa chain V-II region TEW OS=Homo sapiens PE=1 SV=1 | 16.313 | 0.444 | 16.664 | 0.224 | 0.784 | Down | 1.299 | 0.109 | - |
| HEMO_HUMAN | Hemopexin OS=Homo sapiens GN=HPX PE=1 SV=2 | 17.427 | 0.092 | 17.332 | 0.099 | 1.068 | UP | 1.281 | 0.113 | - |
| SPRC_HUMAN | SPARC OS=Homo sapiens GN=SPARC PE=1 SV=1 | 17.323 | 0.147 | 17.167 | 0.170 | 1.115 | UP | 1.274 | 0.114 | - |
| GLU2B_HUMAN-DECOY | GLU2B_HUMAN-DECOY | 18.346 | 0.230 | 18.161 | 0.134 | 1.136 | UP | 1.267 | 0.114 | - |
| LYVE1_HUMAN | Lymphatic vessel endothelial hyaluronic acid receptor 1 OS=Homo sapiens GN=LYVE1 PE=1 SV=2 | 16.056 | 0.180 | 15.901 | 0.145 | 1.113 | UP | 1.206 | 0.130 | - |
| APOA2_HUMAN | Apolipoprotein A-II OS=Homo sapiens GN=APOA2 PE=1 SV=1 | 18.269 | 0.262 | 18.469 | 0.141 | 0.871 | Down | 1.213 | 0.130 | - |
| DSG2_HUMAN | Desmoglein-2 OS=Homo sapiens GN=DSG2 PE=1 SV=2 | 15.521 | 0.158 | 15.378 | 0.147 | 1.104 | UP | 1.197 | 0.131 | - |
| IL1AP_HUMAN | Interleukin-1 receptor accessory protein OS=Homo sapiens GN=IL1RAP PE=1 SV=2 | 16.230 | 0.232 | 16.051 | 0.134 | 1.132 | UP | 1.206 | 0.131 | - |
| MBL2_HUMAN | Mannose-binding protein C OS=Homo sapiens GN=MBL2 PE=1 SV=2 | 17.066 | 0.259 | 16.689 | 0.507 | 1.298 | UP | 1.189 | 0.131 | - |
| APOE_HUMAN | Apolipoprotein E OS=Homo sapiens GN=APOE PE=1 SV=1 | 17.312 | 0.224 | 17.477 | 0.108 | 0.892 | Down | 1.190 | 0.132 | - |
| 1A01_HUMAN (+4) | HLA class I histocompatibility antigen, A-1 alpha chain OS=Homo sapiens GN=HLA-A PE=1 SV=1 | 15.738 | 0.170 | 15.994 | 0.355 | 0.837 | Down | 1.168 | 0.137 | - |
| FA12_HUMAN | Coagulation factor XII OS=Homo sapiens GN=F12 PE=1 SV=3 | 17.857 | 0.368 | 17.567 | 0.258 | 1.223 | UP | 1.149 | 0.143 | - |
| CO6A3_HUMAN | Collagen alpha-3(VI) chain OS=Homo sapiens GN=COL6A3 PE=1 SV=5 | 15.786 | 0.162 | 15.637 | 0.170 | 1.109 | UP | 1.126 | 0.145 | - |
| TRML1_HUMAN | Trem-like transcript 1 protein OS=Homo sapiens GN=TREML1 PE=1 SV=2 | 16.569 | 0.170 | 16.430 | 0.137 | 1.101 | UP | 1.126 | 0.147 | - |
| AFAM_HUMAN | Afamin OS=Homo sapiens GN=AFM PE=1 SV=1 | 17.229 | 0.338 | 16.999 | 0.127 | 1.173 | UP | 1.130 | 0.147 | - |
| PON3_HUMAN | Serum paraoxonase/lactonase 3 OS=Homo sapiens GN=PON3 PE=1 SV=3 | 16.047 | 0.121 | 16.169 | 0.148 | 0.919 | Down | 1.132 | 0.147 | - |
| GPX3_HUMAN | Glutathione peroxidase 3 OS=Homo sapiens GN=GPX3 PE=1 SV=2 | 16.780 | 0.297 | 16.584 | 0.132 | 1.145 | UP | 1.047 | 0.175 | - |
| VASN_HUMAN | Vasorin OS=Homo sapiens GN=VASN PE=1 SV=1 | 16.087 | 0.336 | 15.840 | 0.237 | 1.186 | UP | 1.043 | 0.175 | - |
| CLUS_HUMAN | Clusterin OS=Homo sapiens GN=CLU PE=1 SV=1 | 17.542 | 0.125 | 17.450 | 0.089 | 1.066 | UP | 1.039 | 0.176 | - |
| ALBU_HUMAN | Serum albumin OS=Homo sapiens GN=ALB PE=1 SV=2 | 17.008 | 0.220 | 17.153 | 0.113 | 0.904 | Down | 1.014 | 0.187 | - |
| HPT_HUMAN | Haptoglobin OS=Homo sapiens GN=HP PE=1 SV=1 | 17.280 | 0.747 | 16.806 | 0.337 | 1.389 | UP | 0.994 | 0.195 | - |
| C1QA_HUMAN | Complement C1q subcomponent subunit A OS=Homo sapiens GN=C1QA PE=1 SV=2 | 18.072 | 0.295 | 17.867 | 0.199 | 1.153 | UP | 0.990 | 0.196 | - |
| IBP3_HUMAN | Insulin-like growth factor-binding protein 3 OS=Homo sapiens GN=IGFBP3 PE=1 SV=2 | 16.654 | 0.201 | 16.493 | 0.197 | 1.118 | UP | 0.977 | 0.197 | - |
| KLKB1_HUMAN | Plasma kallikrein OS=Homo sapiens GN=KLKB1 PE=1 SV=1 | 17.313 | 0.214 | 17.444 | 0.080 | 0.913 | Down | 0.983 | 0.197 | - |
| FIBA_HUMAN | Fibrinogen alpha chain OS=Homo sapiens GN=FGA PE=1 SV=2 | 17.609 | 0.347 | 17.388 | 0.174 | 1.166 | UP | 0.970 | 0.198 | - |
| HV302_HUMAN | Ig heavy chain V-III region WEA OS=Homo sapiens PE=1 SV=1 | 16.773 | 0.389 | 17.079 | 0.364 | 0.809 | Down | 0.979 | 0.198 | - |
| FA5_HUMAN | Coagulation factor V OS=Homo sapiens GN=F5 PE=1 SV=4 | 16.401 | 0.130 | 16.309 | 0.096 | 1.066 | UP | 0.972 | 0.198 | - |
| CO5_HUMAN | Complement C5 OS=Homo sapiens GN=C5 PE=1 SV=4 | 16.940 | 0.214 | 16.800 | 0.127 | 1.102 | UP | 0.955 | 0.203 | - |
| HV305_HUMAN | Ig heavy chain V-III region BRO OS=Homo sapiens PE=1 SV=1 | 15.841 | 0.259 | 16.020 | 0.187 | 0.883 | Down | 0.950 | 0.205 | - |
| APOF_HUMAN | Apolipoprotein F OS=Homo sapiens GN=APOF PE=1 SV=2 | 16.418 | 0.251 | 16.569 | 0.111 | 0.901 | Down | 0.929 | 0.215 | - |
| FCGBP_HUMAN | IgGFc-binding protein OS=Homo sapiens GN=FCGBP PE=1 SV=3 | 16.754 | 0.361 | 16.550 | 0.110 | 1.152 | UP | 0.908 | 0.225 | - |
| HPTR_HUMAN | Haptoglobin-related protein OS=Homo sapiens GN=HPR PE=2 SV=2 | 16.952 | 0.270 | 16.758 | 0.242 | 1.144 | UP | 0.896 | 0.230 | - |
| PDIA1_HUMAN | Protein disulfide-isomerase OS=Homo sapiens GN=P4HB PE=1 SV=3 | 15.026 | 0.199 | 14.850 | 0.262 | 1.129 | UP | 0.888 | 0.231 | - |
| COF1_HUMAN | Cofilin-1 OS=Homo sapiens GN=CFL1 PE=1 SV=3 | 16.910 | 0.192 | 16.732 | 0.272 | 1.131 | UP | 0.890 | 0.232 | - |
| APMAP_HUMAN | APMAP_HUMAN | 16.340 | 0.153 | 16.206 | 0.203 | 1.098 | UP | 0.878 | 0.236 | - |
| KV119_HUMAN | Ig kappa chain V-I region Wes OS=Homo sapiens PE=1 SV=1 | 16.823 | 0.274 | 17.008 | 0.221 | 0.880 | Down | 0.868 | 0.239 | - |
| C1QB_HUMAN | Complement C1q subcomponent subunit B OS=Homo sapiens GN=C1QB PE=1 SV=3 | 17.021 | 0.108 | 17.113 | 0.141 | 0.938 | Down | 0.858 | 0.245 | - |
| C1QC_HUMAN | Complement C1q subcomponent subunit C OS=Homo sapiens GN=C1QC PE=1 SV=3 | 17.338 | 0.089 | 17.440 | 0.176 | 0.932 | Down | 0.854 | 0.246 | - |
| VTDB_HUMAN | Vitamin D-binding protein OS=Homo sapiens GN=GC PE=1 SV=1 | 17.560 | 0.312 | 17.387 | 0.125 | 1.128 | UP | 0.849 | 0.247 | - |
| ALS_HUMAN | Insulin-like growth factor-binding protein complex acid labile subunit OS=Homo sapiens GN=IGFALS PE=1 SV=1 | 16.438 | 0.272 | 16.281 | 0.141 | 1.115 | UP | 0.840 | 0.249 | - |
| LCAT_HUMAN | Phosphatidylcholine-sterol acyltransferase OS=Homo sapiens GN=LCAT PE=1 SV=1 | 17.086 | 0.133 | 17.003 | 0.090 | 1.059 | UP | 0.842 | 0.250 | - |
| CBPN_HUMAN | Carboxypeptidase N catalytic chain OS=Homo sapiens GN=CPN1 PE=1 SV=1 | 17.507 | 0.139 | 17.618 | 0.169 | 0.926 | Down | 0.830 | 0.251 | - |
| APOC2_HUMAN | Apolipoprotein C-II OS=Homo sapiens GN=APOC2 PE=1 SV=1 | 18.491 | 0.410 | 18.732 | 0.240 | 0.846 | Down | 0.831 | 0.252 | - |
| ITIH4_HUMAN | Inter-alpha-trypsin inhibitor heavy chain H4 OS=Homo sapiens GN=ITIH4 PE=1 SV=4 | 17.191 | 0.176 | 17.098 | 0.058 | 1.067 | UP | 0.826 | 0.253 | - |
| COL11_HUMAN | Collectin-11 OS=Homo sapiens GN=COLEC11 PE=1 SV=1 | 15.887 | 0.171 | 15.778 | 0.143 | 1.078 | UP | 0.791 | 0.275 | - |
| PXDC2_HUMAN | Plexin domain-containing protein 2 OS=Homo sapiens GN=PLXDC2 PE=1 SV=1 | 15.096 | 0.146 | 14.957 | 0.247 | 1.101 | UP | 0.779 | 0.282 | - |
| AOC3_HUMAN | Membrane primary amine oxidase OS=Homo sapiens GN=AOC3 PE=1 SV=3 | 15.703 | 0.238 | 15.837 | 0.159 | 0.912 | Down | 0.740 | 0.312 | - |
| HV320_HUMAN | Ig heavy chain V-III region GAL OS=Homo sapiens PE=1 SV=1 | 16.883 | 0.257 | 17.029 | 0.187 | 0.904 | Down | 0.725 | 0.322 | - |
| TRFE_HUMAN | Serotransferrin OS=Homo sapiens GN=TF PE=1 SV=3 | 17.701 | 0.190 | 17.593 | 0.145 | 1.078 | UP | 0.711 | 0.330 | - |
| PLSL_HUMAN | Plastin-2 OS=Homo sapiens GN=LCP1 PE=1 SV=6 | 16.060 | 0.256 | 15.901 | 0.251 | 1.116 | UP | 0.694 | 0.343 | - |
| APOC3_HUMAN | Apolipoprotein C-III OS=Homo sapiens GN=APOC3 PE=1 SV=1 | 17.124 | 0.187 | 17.231 | 0.164 | 0.929 | Down | 0.664 | 0.367 | - |
| FBLN3_HUMAN | EGF-containing fibulin-like extracellular matrix protein 1 OS=Homo sapiens GN=EFEMP1 PE=1 SV=2 | 17.048 | 0.190 | 16.958 | 0.092 | 1.064 | UP | 0.658 | 0.370 | - |
| KNG1_HUMAN | Kininogen-1 OS=Homo sapiens GN=KNG1 PE=1 SV=2 | 17.364 | 0.128 | 17.292 | 0.115 | 1.051 | UP | 0.646 | 0.379 | - |
| CO4B_HUMAN | Complement C4-B OS=Homo sapiens GN=C4B PE=1 SV=2 | 17.346 | 0.231 | 17.147 | 0.421 | 1.148 | UP | 0.634 | 0.388 | - |
| GRP78_HUMAN | 78 kDa glucose-regulated protein OS=Homo sapiens GN=HSPA5 PE=1 SV=2 | 16.338 | 0.116 | 16.277 | 0.093 | 1.043 | UP | 0.630 | 0.391 | - |
| HV206_HUMAN | Ig heavy chain V-II region WAH OS=Homo sapiens PE=1 SV=1 | 18.439 | 0.391 | 18.628 | 0.247 | 0.877 | Down | 0.623 | 0.392 | - |
| FHR2_HUMAN | Complement factor H-related protein 2 OS=Homo sapiens GN=CFHR2 PE=1 SV=1 | 17.120 | 0.427 | 16.909 | 0.289 | 1.158 | UP | 0.625 | 0.393 | - |
| NID1_HUMAN | Nidogen-1 OS=Homo sapiens GN=NID1 PE=1 SV=3 | 16.316 | 0.116 | 16.223 | 0.201 | 1.066 | UP | 0.601 | 0.400 | - |
| GPV_HUMAN | Platelet glycoprotein V OS=Homo sapiens GN=GP5 PE=1 SV=1 | 16.531 | 0.163 | 16.444 | 0.143 | 1.062 | UP | 0.606 | 0.400 | - |
| KV301_HUMAN | KV301_HUMAN | 15.647 | 0.305 | 15.777 | 0.110 | 0.914 | Down | 0.608 | 0.402 | - |
| ENOA_HUMAN | Alpha-enolase OS=Homo sapiens GN=ENO1 PE=1 SV=2 | 16.300 | 0.129 | 16.216 | 0.169 | 1.060 | UP | 0.602 | 0.402 | - |
| FCN3_HUMAN | Ficolin-3 OS=Homo sapiens GN=FCN3 PE=1 SV=2 | 17.377 | 0.245 | 17.493 | 0.157 | 0.922 | Down | 0.608 | 0.404 | - |
| POSTN_HUMAN | Periostin OS=Homo sapiens GN=POSTN PE=1 SV=2 | 16.410 | 0.308 | 16.559 | 0.228 | 0.902 | Down | 0.585 | 0.415 | - |
| THBG_HUMAN | Thyroxine-binding globulin OS=Homo sapiens GN=SERPINA7 PE=1 SV=2 | 16.868 | 0.180 | 16.959 | 0.153 | 0.939 | Down | 0.577 | 0.420 | - |
| IGHG2_HUMAN | Ig gamma-2 chain C region OS=Homo sapiens GN=IGHG2 PE=1 SV=2 | 16.199 | 0.633 | 16.480 | 0.369 | 0.823 | Down | 0.574 | 0.421 | - |
| KV105_HUMAN | Ig kappa chain V-I region DEE OS=Homo sapiens PE=1 SV=1 | 15.277 | 0.449 | 15.474 | 0.266 | 0.872 | Down | 0.566 | 0.427 | - |
| IGHD_HUMAN | Ig delta chain C region OS=Homo sapiens GN=IGHD PE=1 SV=2 | 17.198 | 0.706 | 16.879 | 0.490 | 1.247 | UP | 0.549 | 0.443 | - |
| APOL1_HUMAN | Apolipoprotein L1 OS=Homo sapiens GN=APOL1 PE=1 SV=5 | 18.033 | 0.200 | 17.948 | 0.118 | 1.061 | UP | 0.545 | 0.445 | - |
| RET4_HUMAN | Retinol-binding protein 4 OS=Homo sapiens GN=RBP4 PE=1 SV=3 | 16.106 | 0.347 | 15.970 | 0.135 | 1.099 | UP | 0.536 | 0.454 | - |
| PROP_HUMAN | Properdin OS=Homo sapiens GN=CFP PE=1 SV=2 | 17.098 | 0.182 | 17.013 | 0.146 | 1.060 | UP | 0.532 | 0.455 | - |
| LAMP1_HUMAN | LAMP1_HUMAN | 15.017 | 0.179 | 14.908 | 0.244 | 1.078 | UP | 0.528 | 0.456 | - |
| G3P_HUMAN | Glyceraldehyde-3-phosphate dehydrogenase OS=Homo sapiens GN=GAPDH PE=1 SV=3 | 16.511 | 0.212 | 16.406 | 0.206 | 1.076 | UP | 0.523 | 0.457 | - |
| CRAC1_HUMAN | Cartilage acidic protein 1 OS=Homo sapiens GN=CRTAC1 PE=1 SV=2 | 17.927 | 0.197 | 17.851 | 0.078 | 1.054 | UP | 0.523 | 0.459 | - |
| NOE1_HUMAN | Noelin OS=Homo sapiens GN=OLFM1 PE=1 SV=4 | 15.537 | 0.244 | 15.638 | 0.151 | 0.932 | Down | 0.513 | 0.461 | - |
| CYTC_HUMAN | Cystatin-C OS=Homo sapiens GN=CST3 PE=1 SV=1 | 17.328 | 0.128 | 17.271 | 0.097 | 1.040 | UP | 0.514 | 0.463 | - |
| HGFA_HUMAN | Hepatocyte growth factor activator OS=Homo sapiens GN=HGFAC PE=1 SV=1 | 16.303 | 0.155 | 16.369 | 0.107 | 0.956 | Down | 0.504 | 0.471 | - |
| TTHY_HUMAN | Transthyretin OS=Homo sapiens GN=TTR PE=1 SV=1 | 17.531 | 0.113 | 17.478 | 0.111 | 1.038 | UP | 0.486 | 0.488 | - |
| HBA_HUMAN | Hemoglobin subunit alpha OS=Homo sapiens GN=HBA1 PE=1 SV=2 | 16.731 | 0.243 | 16.979 | 0.691 | 0.842 | Down | 0.488 | 0.489 | - |
| LV105_HUMAN | Ig lambda chain V-I region NEWM OS=Homo sapiens PE=1 SV=1 | 17.487 | 0.200 | 17.576 | 0.175 | 0.940 | Down | 0.480 | 0.494 | - |
| MMRN1_HUMAN | Multimerin-1 OS=Homo sapiens GN=MMRN1 PE=1 SV=3 | 16.137 | 0.181 | 16.060 | 0.146 | 1.055 | UP | 0.471 | 0.502 | - |
| IGJ_HUMAN | Immunoglobulin J chain OS=Homo sapiens GN=JCHAIN PE=1 SV=4 | 15.932 | 0.375 | 16.086 | 0.284 | 0.899 | Down | 0.465 | 0.506 | - |
| ITIH1_HUMAN | Inter-alpha-trypsin inhibitor heavy chain H1 OS=Homo sapiens GN=ITIH1 PE=1 SV=3 | 17.207 | 0.092 | 17.246 | 0.078 | 0.973 | Down | 0.459 | 0.506 | - |
| MASP1_HUMAN | Mannan-binding lectin serine protease 1 OS=Homo sapiens GN=MASP1 PE=1 SV=3 | 16.884 | 0.129 | 16.823 | 0.139 | 1.043 | UP | 0.459 | 0.508 | - |
| SEPP1_HUMAN | Selenoprotein P OS=Homo sapiens GN=SEPP1 PE=1 SV=3 | 17.383 | 0.243 | 17.293 | 0.135 | 1.064 | UP | 0.461 | 0.508 | - |
| C1RL_HUMAN | Complement C1r subcomponent-like protein OS=Homo sapiens GN=C1RL PE=1 SV=2 | 17.590 | 0.101 | 17.550 | 0.075 | 1.028 | UP | 0.450 | 0.509 | - |
| KV114_HUMAN | Ig kappa chain V-I region OU OS=Homo sapiens PE=1 SV=1 | 16.620 | 0.204 | 16.694 | 0.114 | 0.950 | Down | 0.452 | 0.509 | - |
| S10A9_HUMAN | Protein S100-A9 OS=Homo sapiens GN=S100A9 PE=1 SV=1 | 17.296 | 0.687 | 17.046 | 0.373 | 1.189 | UP | 0.454 | 0.509 | - |
| SPRL1_HUMAN | SPARC-like protein 1 OS=Homo sapiens GN=SPARCL1 PE=1 SV=2 | 14.701 | 0.397 | 14.913 | 0.556 | 0.863 | Down | 0.438 | 0.510 | - |
| TFR1_HUMAN | Transferrin receptor protein 1 OS=Homo sapiens GN=TFRC PE=1 SV=2 | 16.084 | 0.182 | 16.011 | 0.146 | 1.052 | UP | 0.444 | 0.510 | - |
| HV307_HUMAN | Ig heavy chain V-III region CAM OS=Homo sapiens PE=1 SV=1 | 16.778 | 0.275 | 16.896 | 0.259 | 0.922 | Down | 0.439 | 0.511 | - |
| CO1A1_HUMAN | Collagen alpha-1(I) chain OS=Homo sapiens GN=COL1A1 PE=1 SV=5 | 18.348 | 0.691 | 18.120 | 0.215 | 1.171 | UP | 0.445 | 0.512 | - |
| CO2_HUMAN | Complement C2 OS=Homo sapiens GN=C2 PE=1 SV=2 | 16.631 | 0.169 | 16.692 | 0.098 | 0.959 | Down | 0.440 | 0.513 | - |
| TENX_HUMAN | Tenascin-X OS=Homo sapiens GN=TNXB PE=1 SV=4 | 16.042 | 0.117 | 15.989 | 0.132 | 1.038 | UP | 0.423 | 0.526 | - |
| FINC_HUMAN | Fibronectin OS=Homo sapiens GN=FN1 PE=1 SV=4 | 16.960 | 0.217 | 16.880 | 0.171 | 1.057 | UP | 0.400 | 0.544 | - |
| CADH1_HUMAN | Cadherin-1 OS=Homo sapiens GN=CDH1 PE=1 SV=3 | 16.106 | 0.150 | 16.028 | 0.220 | 1.055 | UP | 0.406 | 0.545 | - |
| PGRP2_HUMAN | N-acetylmuramoyl-L-alanine amidase OS=Homo sapiens GN=PGLYRP2 PE=1 SV=1 | 16.840 | 0.222 | 16.759 | 0.169 | 1.058 | UP | 0.403 | 0.545 | - |
| IGHG3_HUMAN | Ig gamma-3 chain C region OS=Homo sapiens GN=IGHG3 PE=1 SV=2 | 17.918 | 0.621 | 18.121 | 0.324 | 0.869 | Down | 0.401 | 0.545 | - |
| FCN2_HUMAN | Ficolin-2 OS=Homo sapiens GN=FCN2 PE=1 SV=2 | 16.752 | 0.161 | 16.663 | 0.257 | 1.064 | UP | 0.407 | 0.546 | - |
| CO8B_HUMAN | Complement component C8 beta chain OS=Homo sapiens GN=C8B PE=1 SV=3 | 16.862 | 0.223 | 16.786 | 0.147 | 1.055 | UP | 0.396 | 0.547 | - |
| CO7_HUMAN | Complement component C7 OS=Homo sapiens GN=C7 PE=1 SV=2 | 16.766 | 0.130 | 16.720 | 0.093 | 1.032 | UP | 0.392 | 0.550 | - |
| CAH2_HUMAN | Carbonic anhydrase 2 OS=Homo sapiens GN=CA2 PE=1 SV=2 | 16.206 | 0.304 | 16.079 | 0.330 | 1.092 | UP | 0.388 | 0.552 | - |
| APOM_HUMAN | Apolipoprotein M OS=Homo sapiens GN=APOM PE=1 SV=2 | 18.144 | 0.186 | 18.218 | 0.189 | 0.950 | Down | 0.379 | 0.562 | - |
| BLVRB_HUMAN | Flavin reductase (NADPH) OS=Homo sapiens GN=BLVRB PE=1 SV=3 | 16.551 | 0.175 | 16.648 | 0.310 | 0.935 | Down | 0.370 | 0.571 | - |
| ENPP2_HUMAN | Ectonucleotide pyrophosphatase/phosphodiesterase family member 2 OS=Homo sapiens GN=ENPP2 PE=1 SV=3 | 15.488 | 0.232 | 15.407 | 0.199 | 1.058 | UP | 0.359 | 0.578 | - |
| PON1_HUMAN | Serum paraoxonase/arylesterase 1 OS=Homo sapiens GN=PON1 PE=1 SV=3 | 16.763 | 0.238 | 16.842 | 0.177 | 0.947 | Down | 0.360 | 0.580 | - |
| APOD_HUMAN | Apolipoprotein D OS=Homo sapiens GN=APOD PE=1 SV=1 | 17.772 | 0.098 | 17.822 | 0.159 | 0.966 | Down | 0.362 | 0.580 | - |
| CERU_HUMAN | Ceruloplasmin OS=Homo sapiens GN=CP PE=1 SV=1 | 16.750 | 0.087 | 16.791 | 0.134 | 0.972 | Down | 0.346 | 0.595 | - |
| CAD13_HUMAN | Cadherin-13 OS=Homo sapiens GN=CDH13 PE=1 SV=1 | 15.543 | 0.155 | 15.494 | 0.118 | 1.034 | UP | 0.335 | 0.608 | - |
| PTPRJ_HUMAN | Receptor-type tyrosine-protein phosphatase eta OS=Homo sapiens GN=PTPRJ PE=1 SV=3 | 16.240 | 0.220 | 16.179 | 0.124 | 1.043 | UP | 0.320 | 0.619 | - |
| HGFL_HUMAN | Hepatocyte growth factor-like protein OS=Homo sapiens GN=MST1 PE=1 SV=2 | 16.906 | 0.130 | 16.943 | 0.084 | 0.974 | Down | 0.324 | 0.619 | - |
| TENA_HUMAN | Tenascin OS=Homo sapiens GN=TNC PE=1 SV=3 | 16.139 | 0.101 | 16.061 | 0.300 | 1.055 | UP | 0.326 | 0.620 | - |
| CPN2_HUMAN | Carboxypeptidase N subunit 2 OS=Homo sapiens GN=CPN2 PE=1 SV=3 | 16.800 | 0.120 | 16.758 | 0.127 | 1.030 | UP | 0.321 | 0.622 | - |
| ITIH3_HUMAN | Inter-alpha-trypsin inhibitor heavy chain H3 OS=Homo sapiens GN=ITIH3 PE=1 SV=2 | 17.191 | 0.210 | 17.128 | 0.165 | 1.045 | UP | 0.313 | 0.624 | - |
| CXCL7_HUMAN | Platelet basic protein OS=Homo sapiens GN=PPBP PE=1 SV=3 | 17.168 | 0.176 | 17.248 | 0.291 | 0.946 | Down | 0.309 | 0.625 | - |
| HV103_HUMAN | Ig heavy chain V-I region V35 OS=Homo sapiens PE=1 SV=1 | 17.532 | 0.431 | 17.681 | 0.455 | 0.902 | Down | 0.313 | 0.626 | - |
| VCAM1_HUMAN | Vascular cell adhesion protein 1 OS=Homo sapiens GN=VCAM1 PE=1 SV=1 | 16.561 | 0.148 | 16.509 | 0.162 | 1.037 | UP | 0.314 | 0.627 | - |
| CHLE_HUMAN | Cholinesterase OS=Homo sapiens GN=BCHE PE=1 SV=1 | 16.650 | 0.195 | 16.596 | 0.134 | 1.038 | UP | 0.301 | 0.628 | - |
| NCAM1_HUMAN | Neural cell adhesion molecule 1 OS=Homo sapiens GN=NCAM1 PE=1 SV=3 | 16.840 | 0.257 | 16.906 | 0.118 | 0.956 | Down | 0.304 | 0.629 | - |
| OMD_HUMAN | Osteomodulin OS=Homo sapiens GN=OMD PE=1 SV=1 | 16.534 | 0.288 | 16.607 | 0.124 | 0.951 | Down | 0.302 | 0.629 | - |
| CADH5_HUMAN | Cadherin-5 OS=Homo sapiens GN=CDH5 PE=1 SV=5 | 17.258 | 0.163 | 17.213 | 0.108 | 1.031 | UP | 0.296 | 0.630 | - |
| CD5L_HUMAN | CD5 antigen-like OS=Homo sapiens GN=CD5L PE=1 SV=1 | 17.034 | 0.316 | 17.140 | 0.346 | 0.929 | Down | 0.293 | 0.632 | - |
| HEP2_HUMAN | Heparin cofactor 2 OS=Homo sapiens GN=SERPIND1 PE=1 SV=3 | 17.619 | 0.197 | 17.567 | 0.119 | 1.037 | UP | 0.297 | 0.632 | - |
| SAA4_HUMAN | Serum amyloid A-4 protein OS=Homo sapiens GN=SAA4 PE=1 SV=2 | 18.590 | 0.324 | 18.671 | 0.164 | 0.945 | Down | 0.291 | 0.633 | - |
| PIGR_HUMAN | Polymeric immunoglobulin receptor OS=Homo sapiens GN=PIGR PE=1 SV=4 | 16.387 | 0.160 | 16.337 | 0.171 | 1.035 | UP | 0.275 | 0.651 | - |
| DOPO_HUMAN | Dopamine beta-hydroxylase OS=Homo sapiens GN=DBH PE=1 SV=3 | 16.503 | 0.208 | 16.442 | 0.194 | 1.043 | UP | 0.277 | 0.652 | - |
| ECM1_HUMAN | Extracellular matrix protein 1 OS=Homo sapiens GN=ECM1 PE=1 SV=2 | 16.699 | 0.202 | 16.647 | 0.149 | 1.037 | UP | 0.267 | 0.661 | - |
| TETN_HUMAN | Tetranectin OS=Homo sapiens GN=CLEC3B PE=1 SV=3 | 16.303 | 0.374 | 16.222 | 0.139 | 1.058 | UP | 0.259 | 0.664 | - |
| AMPN_HUMAN | Aminopeptidase N OS=Homo sapiens GN=ANPEP PE=1 SV=4 | 16.634 | 0.088 | 16.661 | 0.096 | 0.982 | Down | 0.260 | 0.666 | - |
| PZP_HUMAN | Pregnancy zone protein OS=Homo sapiens GN=PZP PE=1 SV=4 | 14.726 | 0.613 | 14.890 | 0.522 | 0.892 | Down | 0.261 | 0.668 | - |
| HRG_HUMAN | Histidine-rich glycoprotein OS=Homo sapiens GN=HRG PE=1 SV=1 | 16.332 | 0.204 | 16.396 | 0.248 | 0.957 | Down | 0.250 | 0.678 | - |
| A2AP_HUMAN | Alpha-2-antiplasmin OS=Homo sapiens GN=SERPINF2 PE=1 SV=3 | 17.499 | 0.181 | 17.534 | 0.071 | 0.976 | Down | 0.228 | 0.710 | - |
| IGHG4_HUMAN | Ig gamma-4 chain C region OS=Homo sapiens GN=IGHG4 PE=1 SV=1 | 16.899 | 0.627 | 17.039 | 0.469 | 0.908 | Down | 0.222 | 0.718 | - |
| GELS_HUMAN | Gelsolin OS=Homo sapiens GN=GSN PE=1 SV=1 | 16.922 | 0.149 | 16.956 | 0.116 | 0.977 | Down | 0.219 | 0.722 | - |
| PVR_HUMAN | Poliovirus receptor OS=Homo sapiens GN=PVR PE=1 SV=2 | 17.000 | 0.149 | 16.954 | 0.225 | 1.032 | UP | 0.207 | 0.739 | - |
| ICOSL_HUMAN | ICOS ligand OS=Homo sapiens GN=ICOSLG PE=1 SV=2 | 16.884 | 0.169 | 16.830 | 0.289 | 1.038 | UP | 0.199 | 0.744 | - |
| CD109_HUMAN | CD109 antigen OS=Homo sapiens GN=CD109 PE=1 SV=2 | 14.128 | 0.306 | 14.186 | 0.176 | 0.961 | Down | 0.201 | 0.745 | - |
| ANT3_HUMAN | Antithrombin-III OS=Homo sapiens GN=SERPINC1 PE=1 SV=1 | 16.051 | 0.156 | 16.086 | 0.140 | 0.976 | Down | 0.201 | 0.747 | - |
| ATS13_HUMAN | A disintegrin and metalloproteinase with thrombospondin motifs 13 OS=Homo sapiens GN=ADAMTS13 PE=1 SV=1 | 15.840 | 0.143 | 15.803 | 0.185 | 1.026 | UP | 0.191 | 0.757 | - |
| PROS_HUMAN | Vitamin K-dependent protein S OS=Homo sapiens GN=PROS1 PE=1 SV=1 | 17.451 | 0.079 | 17.437 | 0.051 | 1.010 | UP | 0.186 | 0.763 | - |
| FHR1_HUMAN | Complement factor H-related protein 1 OS=Homo sapiens GN=CFHR1 PE=1 SV=2 | 16.734 | 0.191 | 16.767 | 0.105 | 0.978 | Down | 0.178 | 0.770 | - |
| SAMP_HUMAN | Serum amyloid P-component OS=Homo sapiens GN=APCS PE=1 SV=2 | 17.639 | 0.357 | 17.561 | 0.378 | 1.055 | UP | 0.181 | 0.770 | - |
| CNTN1_HUMAN | Contactin-1 OS=Homo sapiens GN=CNTN1 PE=1 SV=1 | 15.364 | 0.113 | 15.396 | 0.180 | 0.979 | Down | 0.176 | 0.772 | - |
| ITIH2_HUMAN | Inter-alpha-trypsin inhibitor heavy chain H2 OS=Homo sapiens GN=ITIH2 PE=1 SV=2 | 17.534 | 0.143 | 17.559 | 0.097 | 0.983 | Down | 0.169 | 0.779 | - |
| KV202_HUMAN | Ig kappa chain V-II region FR OS=Homo sapiens PE=1 SV=1 | 17.606 | 0.333 | 17.690 | 0.492 | 0.943 | Down | 0.170 | 0.780 | - |
| CAH1_HUMAN | Carbonic anhydrase 1 OS=Homo sapiens GN=CA1 PE=1 SV=2 | 16.169 | 0.303 | 16.241 | 0.428 | 0.951 | Down | 0.164 | 0.780 | - |
| IC1_HUMAN | Plasma protease C1 inhibitor OS=Homo sapiens GN=SERPING1 PE=1 SV=2 | 17.247 | 0.170 | 17.219 | 0.106 | 1.019 | UP | 0.166 | 0.781 | - |
| LV302_HUMAN | Ig lambda chain V-III region LOI OS=Homo sapiens PE=1 SV=1 | 17.714 | 0.228 | 17.756 | 0.202 | 0.972 | Down | 0.160 | 0.782 | - |
| CD166_HUMAN | CD166 antigen OS=Homo sapiens GN=ALCAM PE=1 SV=2 | 15.569 | 0.250 | 15.517 | 0.294 | 1.037 | UP | 0.161 | 0.783 | - |
| BGH3_HUMAN | Transforming growth factor-beta-induced protein ig-h3 OS=Homo sapiens GN=TGFBI PE=1 SV=1 | 15.400 | 0.138 | 15.424 | 0.128 | 0.983 | Down | 0.153 | 0.789 | - |
| PLTP_HUMAN | PLTP_HUMAN | 16.011 | 0.304 | 16.057 | 0.173 | 0.969 | Down | 0.154 | 0.790 | - |
| MASP2_HUMAN | Mannan-binding lectin serine protease 2 OS=Homo sapiens GN=MASP2 PE=1 SV=4 | 16.263 | 0.228 | 16.233 | 0.062 | 1.021 | UP | 0.150 | 0.793 | - |
| ZA2G_HUMAN | Zinc-alpha-2-glycoprotein OS=Homo sapiens GN=AZGP1 PE=1 SV=2 | 17.346 | 0.329 | 17.300 | 0.155 | 1.032 | UP | 0.148 | 0.794 | - |
| APOC4_HUMAN | Apolipoprotein C-IV OS=Homo sapiens GN=APOC4 PE=1 SV=1 | 16.949 | 0.453 | 17.022 | 0.389 | 0.950 | Down | 0.144 | 0.798 | - |
| IPSP_HUMAN | Plasma serine protease inhibitor OS=Homo sapiens GN=SERPINA5 PE=1 SV=3 | 16.494 | 0.361 | 16.541 | 0.196 | 0.968 | Down | 0.132 | 0.816 | - |
| ICAM2_HUMAN | Intercellular adhesion molecule 2 OS=Homo sapiens GN=ICAM2 PE=1 SV=2 | 15.786 | 0.177 | 15.760 | 0.136 | 1.018 | UP | 0.134 | 0.817 | - |
| PROZ_HUMAN | Vitamin K-dependent protein Z OS=Homo sapiens GN=PROZ PE=1 SV=2 | 16.554 | 0.258 | 16.516 | 0.281 | 1.027 | UP | 0.117 | 0.842 | - |
| PEPD_HUMAN | Xaa-Pro dipeptidase OS=Homo sapiens GN=PEPD PE=1 SV=3 | 14.106 | 1.898 | 14.294 | 0.458 | 0.877 | Down | 0.110 | 0.853 | - |
| CO6A1_HUMAN | Collagen alpha-1(VI) chain OS=Homo sapiens GN=COL6A1 PE=1 SV=3 | 15.754 | 0.119 | 15.774 | 0.183 | 0.986 | Down | 0.104 | 0.863 | - |
| GGH_HUMAN | Gamma-glutamyl hydrolase OS=Homo sapiens GN=GGH PE=1 SV=2 | 15.942 | 0.188 | 15.969 | 0.246 | 0.982 | Down | 0.097 | 0.874 | - |
| GNPTG_HUMAN | N-acetylglucosamine-1-phosphotransferase subunit gamma OS=Homo sapiens GN=GNPTG PE=1 SV=1 | 14.988 | 0.355 | 15.014 | 0.095 | 0.982 | Down | 0.081 | 0.886 | - |
| F13A_HUMAN | Coagulation factor XIII A chain OS=Homo sapiens GN=F13A1 PE=1 SV=4 | 15.706 | 0.117 | 15.692 | 0.148 | 1.009 | UP | 0.078 | 0.887 | - |
| CD44_HUMAN | CD44 antigen OS=Homo sapiens GN=CD44 PE=1 SV=3 | 16.731 | 0.142 | 16.744 | 0.117 | 0.991 | Down | 0.081 | 0.889 | - |
| KV113_HUMAN | Ig kappa chain V-I region Lay OS=Homo sapiens PE=1 SV=1 | 16.644 | 0.399 | 16.682 | 0.261 | 0.974 | Down | 0.089 | 0.890 | - |
| HBD_HUMAN | Hemoglobin subunit delta OS=Homo sapiens GN=HBD PE=1 SV=2 | 16.566 | 0.216 | 16.526 | 0.500 | 1.028 | UP | 0.082 | 0.890 | - |
| IGKC_HUMAN | Ig kappa chain C region OS=Homo sapiens GN=IGKC PE=1 SV=1 | 15.906 | 0.318 | 15.932 | 0.175 | 0.982 | Down | 0.082 | 0.894 | - |
| PRDX2_HUMAN | Peroxiredoxin-2 OS=Homo sapiens GN=PRDX2 PE=1 SV=5 | 16.857 | 0.229 | 16.896 | 0.458 | 0.973 | Down | 0.085 | 0.894 | - |
| ATRN_HUMAN | ATRN_HUMAN | 16.962 | 0.109 | 16.950 | 0.123 | 1.009 | UP | 0.083 | 0.895 | - |
| APOA_HUMAN | Apolipoprotein(a) OS=Homo sapiens GN=LPA PE=1 SV=1 | 17.259 | 0.326 | 17.228 | 0.423 | 1.022 | UP | 0.064 | 0.916 | - |
| NCHL1_HUMAN | Neural cell adhesion molecule L1-like protein OS=Homo sapiens GN=CHL1 PE=1 SV=4 | 15.801 | 0.181 | 15.789 | 0.156 | 1.009 | UP | 0.055 | 0.927 | - |
| PCYOX_HUMAN | Prenylcysteine oxidase 1 OS=Homo sapiens GN=PCYOX1 PE=1 SV=3 | 15.851 | 0.226 | 15.837 | 0.165 | 1.010 | UP | 0.056 | 0.929 | - |
| COMP_HUMAN | Cartilage oligomeric matrix protein OS=Homo sapiens GN=COMP PE=1 SV=2 | 14.950 | 0.132 | 14.939 | 0.199 | 1.008 | UP | 0.050 | 0.936 | - |
| CFAB_HUMAN | Complement factor B OS=Homo sapiens GN=CFB PE=1 SV=2 | 17.560 | 0.292 | 17.576 | 0.189 | 0.989 | Down | 0.048 | 0.937 | - |
| LDHB_HUMAN | L-lactate dehydrogenase B chain OS=Homo sapiens GN=LDHB PE=1 SV=2 | 16.528 | 0.135 | 16.519 | 0.168 | 1.006 | UP | 0.044 | 0.942 | - |
| MUC18_HUMAN | Cell surface glycoprotein MUC18 OS=Homo sapiens GN=MCAM PE=1 SV=2 | 15.810 | 0.169 | 15.800 | 0.221 | 1.007 | UP | 0.038 | 0.952 | - |
| KV203_HUMAN | Ig kappa chain V-II region MIL OS=Homo sapiens PE=1 SV=1 | 13.599 | 0.249 | 13.578 | 0.571 | 1.015 | UP | 0.036 | 0.954 | - |
| LUM_HUMAN | Lumican OS=Homo sapiens GN=LUM PE=1 SV=2 | 17.074 | 0.306 | 17.066 | 0.095 | 1.006 | UP | 0.029 | 0.956 | - |
| KAIN_HUMAN | Kallistatin OS=Homo sapiens GN=SERPINA4 PE=1 SV=3 | 15.531 | 0.482 | 15.516 | 0.147 | 1.011 | UP | 0.033 | 0.959 | - |
| ICAM1_HUMAN | Intercellular adhesion molecule 1 OS=Homo sapiens GN=ICAM1 PE=1 SV=2 | 16.063 | 0.111 | 16.069 | 0.166 | 0.996 | Down | 0.029 | 0.959 | - |
| BST1_HUMAN | ADP-ribosyl cyclase/cyclic ADP-ribose hydrolase 2 OS=Homo sapiens GN=BST1 PE=1 SV=2 | 15.482 | 0.264 | 15.470 | 0.334 | 1.009 | UP | 0.030 | 0.960 | - |
| PI16_HUMAN | Peptidase inhibitor 16 OS=Homo sapiens GN=PI16 PE=1 SV=1 | 15.367 | 0.273 | 15.373 | 0.192 | 0.995 | Down | 0.021 | 0.968 | - |
| CETP_HUMAN | Cholesteryl ester transfer protein OS=Homo sapiens GN=CETP PE=1 SV=2 | 17.120 | 0.193 | 17.114 | 0.229 | 1.004 | Up | 0.019 | 0.968 | - |
| FETUB_HUMAN | Fetuin-B OS=Homo sapiens GN=FETUB PE=1 SV=2 | 16.016 | 0.199 | 16.010 | 0.187 | 1.004 | Up | 0.021 | 0.971 | - |
| MMP2_HUMAN | 72 kDa type IV collagenase OS=Homo sapiens GN=MMP2 PE=1 SV=2 | 14.796 | 0.468 | 14.790 | 0.161 | 1.004 | Up | 0.012 | 0.984 | - |
| B2MG_HUMAN | Beta-2-microglobulin OS=Homo sapiens GN=B2M PE=1 SV=1 | 17.447 | 0.321 | 17.448 | 0.111 | 0.999 | Down | 0.003 | 0.994 | - |
| FA11_HUMAN | Coagulation factor XI OS=Homo sapiens GN=F11 PE=1 SV=1 | 16.554 | 0.213 | 16.553 | 0.129 | 1.001 | Up | 0.005 | 0.997 | - |
| HBB_HUMAN | Hemoglobin subunit beta OS=Homo sapiens GN=HBB PE=1 SV=2 | 16.207 | 0.334 | 16.209 | 0.540 | 0.998 | Down | 0.004 | 0.997 | - |
