# Supplementary material for: Comparative analysis of serum proteome in congenital scoliosis patients with TBX6 haploinsufficiency – a first report pointing to lipid metabolism
Source: J Cell Mol Med. 2017 Sep 25;22(1):533–45. doi: 10.1111/jcmm.13341 (PMC5742745; doi:10.1111/jcmm.13341)
Supplement: Supplementary file 2 — Table S2 Network analysis results generated by IPA software. [file JCMM-22-533-s002.doc]

# Comparative analysis of serum proteome in congenital scoliosis patients with *TBX6* haploinsufficiency- a first report pointing to lipid metabolism

Qiankun Zhu^1﹟^, Nan Wu^1, 2, 3﹟^, Gang Liu^1, 2, 3^, Yangzhong Zhou^4^, Sen Liu^1^, Jun Chen^5^, Jiaqi Liu^1^, Yuzhi Zuo^1^, Zhenlei Liu^7^, Weisheng Chen^1^, Yixin Chen^1^, Jia Chen^1^, Mao Lin^1^, Yanxue Zhao^1^, Yang Yang^1^, Shensgru Wang^1^, Xu Yang^1^, Yufen Ma^1^, Jian Wang^8^, Xiaoli Chen^9^, Jianguo Zhang^1^, Jianxiong Shen^1^, Zhihong Wu ^2,3,6*^, Guixing Qiu^1, 2, 3*^

1. Department of Orthopedic Surgery, Peking Union Medical College Hospital, Peking Union Medical College and Chinese Academy of Medical Sciences, Beijing, China
2. Beijing Key Laboratory for Genetic Research of Skeletal Deformity, China
3. Research Center of Orthopedics/Rare Disease, Chinese Academy of Medical Sciences, Beijing, China
4. Tsinghua University Medical School, Beijing, China
5. Department of Pathology, Beijing Ditan Hospital, Capital Medical University, Beijing, China
6. Department of Central Laboratory, Peking Union Medical College Hospital, Peking Union Medical College and Chinese Academy of Medical Sciences, Beijing, China
7. Department of Neurosurgery, Xuanwu Hospital, Capital Medical University, Beijing, China
8. Department of Medical Genetics, Molecular Diagnostic Laboratory, Shanghai Children's Medical Center, Shanghai Jiaotong University School of Medicine, Shanghai, China
9. Department of Medical Genetics, Beijing Municipal Key Laboratory of Child Development and Nutriomics, Capital Institute of Pediatrics, Beijing, China

*Correspondence to: Dr. Guixing Qiu, Department of Orthopedic Surgery, Peking Union Medical College Hospital; Beijing Key Laboratory for Genetic Research of Skeletal Deformity; Research Center of Orthopedics/Rare Disease, Chinese Academy of Medical Sciences, No.1 Shuaifuyuan, Beijing, China. Tel: +8601069152809, E-mail: [qiuguixingpumch@126.com](mailto:qiuguixingpumch@126.com). Zhihong Wu, Beijing Key Laboratory for Genetic Research of Skeletal Deformity, Research Center of Orthopedics/Rare Disease, Department of Central Laboratory, Peking Union Medical College Hospital, Peking Union Medical College and Chinese Academy of Medical Sciences, No.1 Shuaifuyuan, Beijing, China. Tel: +8601069154259, E-mail: [orthoscience@126.com](mailto:orthoscience@126.com).

Guixing Qiu and Zhihong Wu are co-corresponding authors.

The authors declare that there is no conflict of interest.

﹟These authors contributed equally to this work.

| ID | Molecules in Network | Score | Focus Molecules | Top Diseases and Functions |
| --- | --- | --- | --- | --- |
| 1 | 26s Proteasome,ADCYAP1,AGER,C9,C4A/C4B,COL18A1,CRP,CRYAB,ERK1/2,FGFR2,IGHG1,IL6,IL17D,Jnk,KRT1,KRT10,LBP,mir-224,miR-224-5p (miRNAs w/seed AAGUCAC),MMP9,ORM1,P38 MAPK,PPARG,PRKCG,RELA,S100A2,S100A12,SAA1,SAA2,SERPINA1,SHBG,TAGLN2,TP53,TP63,VWF | 37 | 15 | Cellular Movement, Hematological System Development and Function, Immune Cell Trafficking |
| 2 | CKM,EPAS1 | 3 | 1 | Respiratory Disease, Cardiac Hypertrophy, Cardiovascular Disease |
| 3 | CDK5,LRG1 | 3 | 1 | Cardiovascular Disease, Cell Morphology, Neurological Disease |
| 4 | APOC1,ERG | 3 | 1 | Cellular Development, Cellular Function and Maintenance, Cellular Growth and Proliferation |

Supplementary Table 2
