# Supplementary material for: Comparative analysis of serum proteome in congenital scoliosis patients with TBX6 haploinsufficiency – a first report pointing to lipid metabolism
Source: J Cell Mol Med. 2017 Sep 25;22(1):533–45. doi: 10.1111/jcmm.13341 (PMC5742745; doi:10.1111/jcmm.13341)
Supplement: Supplementary file 3 — Table S3 Ingenuity canonical pathways assigned by IPA software. [file JCMM-22-533-s003.doc]

# Comparative analysis of serum proteome in congenital scoliosis patients with *TBX6* haploinsufficiency- a first report pointing to lipid metabolism

Qiankun Zhu^1﹟^, Nan Wu^1, 2, 3﹟^, Gang Liu^1, 2, 3^, Yangzhong Zhou^4^, Sen Liu^1^, Jun Chen^5^, Jiaqi Liu^1^, Yuzhi Zuo^1^, Zhenlei Liu^7^, Weisheng Chen^1^, Yixin Chen^1^, Jia Chen^1^, Mao Lin^1^, Yanxue Zhao^1^, Yang Yang^1^, Shensgru Wang^1^, Xu Yang^1^, Yufen Ma^1^, Jian Wang^8^, Xiaoli Chen^9^, Jianguo Zhang^1^, Jianxiong Shen^1^, Zhihong Wu ^2,3,6*^, Guixing Qiu^1, 2, 3*^

1. Department of Orthopedic Surgery, Peking Union Medical College Hospital, Peking Union Medical College and Chinese Academy of Medical Sciences, Beijing, China
2. Beijing Key Laboratory for Genetic Research of Skeletal Deformity, China
3. Research Center of Orthopedics/Rare Disease, Chinese Academy of Medical Sciences, Beijing, China
4. Tsinghua University Medical School, Beijing, China
5. Department of Pathology, Beijing Ditan Hospital, Capital Medical University, Beijing, China
6. Department of Central Laboratory, Peking Union Medical College Hospital, Peking Union Medical College and Chinese Academy of Medical Sciences, Beijing, China
7. Department of Neurosurgery, Xuanwu Hospital, Capital Medical University, Beijing, China
8. Department of Medical Genetics, Molecular Diagnostic Laboratory, Shanghai Children's Medical Center, Shanghai Jiaotong University School of Medicine, Shanghai, China
9. Department of Medical Genetics, Beijing Municipal Key Laboratory of Child Development and Nutriomics, Capital Institute of Pediatrics, Beijing, China

*Correspondence to: Dr. Guixing Qiu, Department of Orthopedic Surgery, Peking Union Medical College Hospital; Beijing Key Laboratory for Genetic Research of Skeletal Deformity; Research Center of Orthopedics/Rare Disease, Chinese Academy of Medical Sciences, No.1 Shuaifuyuan, Beijing, China. Tel: +8601069152809, E-mail: [qiuguixingpumch@126.com](mailto:qiuguixingpumch@126.com). Zhihong Wu, Beijing Key Laboratory for Genetic Research of Skeletal Deformity, Research Center of Orthopedics/Rare Disease, Department of Central Laboratory, Peking Union Medical College Hospital, Peking Union Medical College and Chinese Academy of Medical Sciences, No.1 Shuaifuyuan, Beijing, China. Tel: +8601069154259, E-mail: [orthoscience@126.com](mailto:orthoscience@126.com).

Guixing Qiu and Zhihong Wu are co-corresponding authors.

The authors declare that there is no conflict of interest.

﹟These authors contributed equally to this work.

| Ingenuity Canonical Pathways | -log(p-value) | Ratio | z-score | Molecules |
| --- | --- | --- | --- | --- |
| LXR/RXR Activation | 1.47E01 | 7.44E-02 | 1.000 | C4A/C4B,ORM1,SAA1,C9,APOC1,SERPINA1,LBP,SAA2,MMP9 |
| Acute Phase Response Signaling | 1.34E01 | 5.36E-02 | NaN | C4A/C4B,ORM1,SAA1,C9,CRP,VWF,SERPINA1,LBP,SAA2 |
| FXR/RXR Activation | 1.05E01 | 5.6E-02 | NaN | C4A/C4B,ORM1,SAA1,C9,APOC1,SERPINA1,SAA2 |
| Atherosclerosis Signaling | 5.13E00 | 3.23E-02 | NaN | ORM1,APOC1,SERPINA1,MMP9 |
| IL-12 Signaling and Production in Macrophages | 3.37E00 | 2.08E-02 | NaN | ORM1,APOC1,SERPINA1 |
| Coagulation System | 3.22E00 | 5.71E-02 | NaN | VWF,SERPINA1 |
| Complement System | 3.2E00 | 5.56E-02 | NaN | C4A/C4B,C9 |
| Production of Nitric Oxide and Reactive Oxygen Species in Macrophages | 3E00 | 1.56E-02 | NaN | ORM1,APOC1,SERPINA1 |
| Clathrin-mediated Endocytosis Signaling | 2.98E00 | 1.53E-02 | NaN | ORM1,APOC1,SERPINA1 |
| Creatine-phosphate Biosynthesis | 2.28E00 | 2E-01 | NaN | CKM |
| IL-6 Signaling | 2.12E00 | 1.57E-02 | NaN | CRP,LBP |
| Airway Pathology in Chronic Obstructive Pulmonary Disease | 2.08E00 | 1.25E-01 | NaN | MMP9 |
| Hepatic Fibrosis / Hepatic Stellate Cell Activation | 1.82E00 | 1.1E-02 | NaN | LBP,MMP9 |
| LPS/IL-1 Mediated Inhibition of RXR Function | 1.71E00 | 9.62E-03 | NaN | APOC1,LBP |
| Systemic Lupus Erythematosus Signaling | 1.67E00 | 9.22E-03 | NaN | C9,IGHG1 |
| Inhibition of Angiogenesis by TSP1 | 1.48E00 | 3.12E-02 | NaN | MMP9 |
| Inhibition of Matrix Metalloproteases | 1.41E00 | 2.63E-02 | NaN | MMP9 |
| Neuroprotective Role of THOP1 in Alzheimer's Disease | 1.39E00 | 2.5E-02 | NaN | MMP9 |
| Autoimmune Thyroid Disease Signaling | 1.37E00 | 2.38E-02 | NaN | IGHG1 |
| Hematopoiesis from Pluripotent Stem Cells | 1.37E00 | 2.38E-02 | NaN | IGHG1 |
| iNOS Signaling | 1.36E00 | 2.33E-02 | NaN | LBP |
| Primary Immunodeficiency Signaling | 1.35E00 | 2.27E-02 | NaN | IGHG1 |
| Allograft Rejection Signaling | 1.31E00 | 2.08E-02 | NaN | IGHG1 |
| IL-10 Signaling | 1.16E00 | 1.47E-02 | NaN | LBP |
| Glioma Invasiveness Signaling | 1.15E00 | 1.43E-02 | NaN | MMP9 |
| Toll-like Receptor Signaling | 1.13E00 | 1.37E-02 | NaN | LBP |
| Communication between Innate and Adaptive Immune Cells | 1.09E00 | 1.22E-02 | NaN | IGHG1 |
| IL-17 Signaling | 1.07E00 | 1.18E-02 | NaN | CRP |
| LPS-stimulated MAPK Signaling | 1.07E00 | 1.16E-02 | NaN | LBP |
| Bladder Cancer Signaling | 1.07E00 | 1.16E-02 | NaN | MMP9 |
| Corticotropin Releasing Hormone Signaling | 9.75E-01 | 9.35E-03 | NaN | KRT1 |
| Androgen Signaling | 9.64E-01 | 9.09E-03 | NaN | SHBG |
| HIF1α Signaling | 9.53E-01 | 8.85E-03 | NaN | MMP9 |
| phagosome formation | 9.45E-01 | 8.7E-03 | NaN | IGHG1 |
| Pancreatic Adenocarcinoma Signaling | 9.35E-01 | 8.47E-03 | NaN | MMP9 |
| Ovarian Cancer Signaling | 8.57E-01 | 6.99E-03 | NaN | MMP9 |
| Relaxin Signaling | 8.46E-01 | 6.8E-03 | NaN | MMP9 |
| Hepatic Cholestasis | 8.19E-01 | 6.37E-03 | NaN | LBP |
| Granulocyte Adhesion and Diapedesis | 7.99E-01 | 6.06E-03 | NaN | MMP9 |
| Agranulocyte Adhesion and Diapedesis | 7.76E-01 | 5.71E-03 | NaN | MMP9 |
| Dendritic Cell Maturation | 7.6E-01 | 5.49E-03 | NaN | IGHG1 |
| B Cell Receptor Signaling | 7.6E-01 | 5.49E-03 | NaN | IGHG1 |
| Regulation of the Epithelial-Mesenchymal Transition Pathway | 7.5E-01 | 5.35E-03 | NaN | MMP9 |
| AMPK Signaling | 7.47E-01 | 5.32E-03 | NaN | CKM |
| ILK Signaling | 7.39E-01 | 5.21E-03 | NaN | MMP9 |
| IL-8 Signaling | 7.31E-01 | 5.1E-03 | NaN | MMP9 |
| Leukocyte Extravasation Signaling | 7.13E-01 | 4.88E-03 | NaN | MMP9 |
| Actin Cytoskeleton Signaling | 6.82E-01 | 4.5E-03 | NaN | LBP |
| Phospholipase C Signaling | 6.67E-01 | 4.33E-03 | NaN | IGHG1 |
| Colorectal Cancer Metastasis Signaling | 6.49E-01 | 4.13E-03 | NaN | MMP9 |
| Role of Macrophages, Fibroblasts and Endothelial Cells in Rheumatoid Arthritis | 5.68E-01 | 3.33E-03 | NaN | IGHG1 |
| Axonal Guidance Signaling | 4.29E-01 | 2.27E-03 | NaN | MMP9 |

Supplementary Table 3
