# Supplementary material for: Comparative analysis of serum proteome in congenital scoliosis patients with TBX6 haploinsufficiency – a first report pointing to lipid metabolism
Source: J Cell Mol Med. 2017 Sep 25;22(1):533–45. doi: 10.1111/jcmm.13341 (PMC5742745; doi:10.1111/jcmm.13341)
Supplement: Supplementary file 4 — Table S4 The separated proteins in cluster 61 and cluster 62 by HCA. [file JCMM-22-533-s004.doc]

# Comparative analysis of serum proteome in congenital scoliosis patients with *TBX6* haploinsufficiency- a first report pointing to lipid metabolism

Qiankun Zhu^1﹟^, Nan Wu^1, 2, 3﹟^, Gang Liu^1, 2, 3^, Yangzhong Zhou^4^, Sen Liu^1^, Jun Chen^5^, Jiaqi Liu^1^, Yuzhi Zuo^1^, Zhenlei Liu^7^, Weisheng Chen^1^, Yixin Chen^1^, Jia Chen^1^, Mao Lin^1^, Yanxue Zhao^1^, Yang Yang^1^, Shensgru Wang^1^, Xu Yang^1^, Yufen Ma^1^, Jian Wang^8^, Xiaoli Chen^9^, Jianguo Zhang^1^, Jianxiong Shen^1^, Zhihong Wu ^2,3,6*^, Guixing Qiu^1, 2, 3*^

1. Department of Orthopedic Surgery, Peking Union Medical College Hospital, Peking Union Medical College and Chinese Academy of Medical Sciences, Beijing, China
2. Beijing Key Laboratory for Genetic Research of Skeletal Deformity, China
3. Research Center of Orthopedics/Rare Disease, Chinese Academy of Medical Sciences, Beijing, China
4. Tsinghua University Medical School, Beijing, China
5. Department of Pathology, Beijing Ditan Hospital, Capital Medical University, Beijing, China
6. Department of Central Laboratory, Peking Union Medical College Hospital, Peking Union Medical College and Chinese Academy of Medical Sciences, Beijing, China
7. Department of Neurosurgery, Xuanwu Hospital, Capital Medical University, Beijing, China
8. Department of Medical Genetics, Molecular Diagnostic Laboratory, Shanghai Children's Medical Center, Shanghai Jiaotong University School of Medicine, Shanghai, China
9. Department of Medical Genetics, Beijing Municipal Key Laboratory of Child Development and Nutriomics, Capital Institute of Pediatrics, Beijing, China

*Correspondence to: Dr. Guixing Qiu, Department of Orthopedic Surgery, Peking Union Medical College Hospital; Beijing Key Laboratory for Genetic Research of Skeletal Deformity; Research Center of Orthopedics/Rare Disease, Chinese Academy of Medical Sciences, No.1 Shuaifuyuan, Beijing, China. Tel: +8601069152809, E-mail: [qiuguixingpumch@126.com](mailto:qiuguixingpumch@126.com). Zhihong Wu, Beijing Key Laboratory for Genetic Research of Skeletal Deformity, Research Center of Orthopedics/Rare Disease, Department of Central Laboratory, Peking Union Medical College Hospital, Peking Union Medical College and Chinese Academy of Medical Sciences, No.1 Shuaifuyuan, Beijing, China. Tel: +8601069154259, E-mail: [orthoscience@126.com](mailto:orthoscience@126.com).

Guixing Qiu and Zhihong Wu are co-corresponding authors.

The authors declare that there is no conflict of interest.

﹟These authors contributed equally to this work.

| C: Cluster | T: Accession Number | T: Identified Proteins |
| --- | --- | --- |
| Cluster 61 | SHBG_HUMAN | Sex hormone-binding globulin OS=Homo sapiens GN=SHBG PE=1 SV=2 |
| Cluster 61 | APOA4_HUMAN | Apolipoprotein A-IV OS=Homo sapiens GN=APOA4 PE=1 SV=3 |
| Cluster 61 | APOC1_HUMAN | Apolipoprotein C-I OS=Homo sapiens GN=APOC1 PE=1 SV=1 |
| Cluster 61 | APOA1_HUMAN | Apolipoprotein A-I OS=Homo sapiens GN=APOA1 PE=1 SV=1 |
| Cluster 61 | K22E_HUMAN | Keratin, type II cytoskeletal 2 epidermal OS=Homo sapiens GN=KRT2 PE=1 SV=2 |
| Cluster 61 | K2C1_HUMAN | Keratin, type II cytoskeletal 1 OS=Homo sapiens GN=KRT1 PE=1 SV=6 |
| Cluster 61 | K1C10_HUMAN | Keratin, type I cytoskeletal 10 OS=Homo sapiens GN=KRT10 PE=1 SV=6 |
| Cluster 61 | HBG2_HUMAN | Hemoglobin subunit gamma-2 OS=Homo sapiens GN=HBG2 PE=1 SV=2 |
| Cluster 61 | IGHM_HUMAN | Ig mu chain C region OS=Homo sapiens GN=IGHM PE=1 SV=3 |
| Cluster 61 | APOB_HUMAN | Apolipoprotein B-100 OS=Homo sapiens GN=APOB PE=1 SV=2 |
| Cluster 61 | IGHG1_HUMAN | Ig gamma-1 chain C region OS=Homo sapiens GN=IGHG1 PE=1 SV=1 |
| Cluster 61 | HV102_HUMAN | Ig heavy chain V-I region HG3 OS=Homo sapiens PE=3 SV=1 |
| Cluster 61 | LV301_HUMAN | Ig lambda chain V-III region SH OS=Homo sapiens PE=1 SV=1 |
| Cluster 61 | KV402_HUMAN | Ig kappa chain V-IV region Len OS=Homo sapiens PE=1 SV=2 |
| Cluster 61 | HV303_HUMAN | Ig heavy chain V-III region 23 OS=Homo sapiens GN=IGHV3-23 PE=1 SV=2 |
| Cluster 61 | LV106_HUMAN | Ig lambda chain V-I region WAH OS=Homo sapiens PE=1 SV=1 |
| Cluster 62 | IGHA1_HUMAN | Ig alpha-1 chain C region OS=Homo sapiens GN=IGHA1 PE=1 SV=2 |
| Cluster 62 | PLF4_HUMAN | Platelet factor 4 OS=Homo sapiens GN=PF4 PE=1 SV=2 |
| Cluster 62 | FHR3_HUMAN | Complement factor H-related protein 3 OS=Homo sapiens GN=CFHR3 PE=1 SV=2 |
| Cluster 62 | GP1BA_HUMAN | Platelet glycoprotein Ib alpha chain OS=Homo sapiens GN=GP1BA PE=1 SV=2 |
| Cluster 62 | CNDP1_HUMAN | Beta-Ala-His dipeptidase OS=Homo sapiens GN=CNDP1 PE=1 SV=4 |
| Cluster 62 | TAGL2_HUMAN | Transgelin-2 OS=Homo sapiens GN=TAGLN2 PE=1 SV=3 |
| Cluster 62 | TSP1_HUMAN | Thrombospondin-1 OS=Homo sapiens GN=THBS1 PE=1 SV=2 |
| Cluster 62 | TLN1_HUMAN | TLN1_HUMAN |
| Cluster 62 | LG3BP_HUMAN | Galectin-3-binding protein OS=Homo sapiens GN=LGALS3BP PE=1 SV=1 |
| Cluster 62 | CO4A_HUMAN | Complement C4-A OS=Homo sapiens GN=C4A PE=1 SV=2 |
| Cluster 62 | ZPI_HUMAN | Protein Z-dependent protease inhibitor OS=Homo sapiens GN=SERPINA10 PE=1 SV=1 |
| Cluster 62 | PROF1_HUMAN | Profilin-1 OS=Homo sapiens GN=PFN1 PE=1 SV=2 |
| Cluster 62 | ACTB_HUMAN (+1) | Actin, cytoplasmic 1 OS=Homo sapiens GN=ACTB PE=1 SV=1 |
| Cluster 62 | NRP1_HUMAN | Neuropilin-1 OS=Homo sapiens GN=NRP1 PE=1 SV=3 |
| Cluster 62 | FA10_HUMAN | Coagulation factor X OS=Homo sapiens GN=F10 PE=1 SV=2 |
| Cluster 62 | PROC_HUMAN | Vitamin K-dependent protein C OS=Homo sapiens GN=PROC PE=1 SV=1 |
| Cluster 62 | PEDF_HUMAN | Pigment epithelium-derived factor OS=Homo sapiens GN=SERPINF1 PE=1 SV=4 |
| Cluster 62 | VTNC_HUMAN | Vitronectin OS=Homo sapiens GN=VTN PE=1 SV=1 |
| Cluster 62 | CO3_HUMAN | Complement C3 OS=Homo sapiens GN=C3 PE=1 SV=2 |
| Cluster 62 | C4BPA_HUMAN | C4b-binding protein alpha chain OS=Homo sapiens GN=C4BPA PE=1 SV=2 |
| Cluster 62 | MA1A1_HUMAN | Mannosyl-oligosaccharide 1,2-alpha-mannosidase IA OS=Homo sapiens GN=MAN1A1 PE=1 SV=3 |
| Cluster 62 | KCRM_HUMAN | Creatine kinase M-type OS=Homo sapiens GN=CKM PE=1 SV=2 |
| Cluster 62 | CD14_HUMAN | Monocyte differentiation antigen CD14 OS=Homo sapiens GN=CD14 PE=1 SV=2 |
| Cluster 62 | LBP_HUMAN | Lipopolysaccharide-binding protein OS=Homo sapiens GN=LBP PE=1 SV=3 |
| Cluster 62 | VWF_HUMAN | von Willebrand factor OS=Homo sapiens GN=VWF PE=1 SV=4 |
| Cluster 62 | F13B_HUMAN | Coagulation factor XIII B chain OS=Homo sapiens GN=F13B PE=1 SV=3 |
| Cluster 62 | MMP9_HUMAN | Matrix metalloproteinase-9 OS=Homo sapiens GN=MMP9 PE=1 SV=3 |
| Cluster 62 | C1S_HUMAN | Complement C1s subcomponent OS=Homo sapiens GN=C1S PE=1 SV=1 |
| Cluster 62 | CFAI_HUMAN | Complement factor I OS=Homo sapiens GN=CFI PE=1 SV=2 |
| Cluster 62 | C1R_HUMAN | Complement C1r subcomponent OS=Homo sapiens GN=C1R PE=1 SV=2 |
| Cluster 62 | PLMN_HUMAN | Plasminogen OS=Homo sapiens GN=PLG PE=1 SV=2 |
| Cluster 62 | HABP2_HUMAN | Hyaluronan-binding protein 2 OS=Homo sapiens GN=HABP2 PE=1 SV=1 |
| Cluster 62 | FA9_HUMAN | Coagulation factor IX OS=Homo sapiens GN=F9 PE=1 SV=2 |
| Cluster 62 | CO6_HUMAN | Complement component C6 OS=Homo sapiens GN=C6 PE=1 SV=3 |
| Cluster 62 | APOH_HUMAN | Beta-2-glycoprotein 1 OS=Homo sapiens GN=APOH PE=1 SV=3 |
| Cluster 62 | FBLN1_HUMAN | Fibulin-1 OS=Homo sapiens GN=FBLN1 PE=1 SV=4 |
| Cluster 62 | AMBP_HUMAN | Protein AMBP OS=Homo sapiens GN=AMBP PE=1 SV=1 |
| Cluster 62 | OAF_HUMAN | Out at first protein homolog OS=Homo sapiens GN=OAF PE=2 SV=1 |
| Cluster 62 | SAA1_HUMAN | Serum amyloid A-1 protein OS=Homo sapiens GN=SAA1 PE=1 SV=1 |
| Cluster 62 | SAA2_HUMAN | Serum amyloid A-2 protein OS=Homo sapiens GN=SAA2 PE=1 SV=1 |
| Cluster 62 | A2GL_HUMAN | Leucine-rich alpha-2-glycoprotein OS=Homo sapiens GN=LRG1 PE=1 SV=2 |
| Cluster 62 | A1AT_HUMAN | Alpha-1-antitrypsin OS=Homo sapiens GN=SERPINA1 PE=1 SV=3 |
| Cluster 62 | CO8G_HUMAN | Complement component C8 gamma chain OS=Homo sapiens GN=C8G PE=1 SV=3 |
| Cluster 62 | CO8A_HUMAN | Complement component C8 alpha chain OS=Homo sapiens GN=C8A PE=1 SV=2 |
| Cluster 62 | C4BPB_HUMAN | C4BPB_HUMAN |
| Cluster 62 | A1AG2_HUMAN | Alpha-1-acid glycoprotein 2 OS=Homo sapiens GN=ORM2 PE=1 SV=2 |
| Cluster 62 | CRP_HUMAN | C-reactive protein OS=Homo sapiens GN=CRP PE=1 SV=1 |
| Cluster 62 | CO9_HUMAN | Complement component C9 OS=Homo sapiens GN=C9 PE=1 SV=2 |
| Cluster 62 | A1AG1_HUMAN | Alpha-1-acid glycoprotein 1 OS=Homo sapiens GN=ORM1 PE=1 SV=1 |

Supplementary Table 4
